# Determining the Impact of Opioid Policy on Substance Use and Mental Health–Related Harms: Protocol for a Data Linkage Study

**DOI:** 10.2196/51825

**Published:** 2023-10-17

**Authors:** Ting Xia, Louisa Picco, Samanta Lalic, Rachelle Buchbinder, J Simon Bell, Nadine E Andrew, Dan I Lubman, Christopher Pearce, Suzanne Nielsen

**Affiliations:** 1 Monash Addiction Research Centre Eastern Health Clinical School Monash University Frankston Australia; 2 Centre for Medicine Use and Safety Faculty of Pharmacy and Pharmaceutical Sciences Monash University Melbourne Australia; 3 Pharmacy Department Monash Health Clayton Australia; 4 Musculoskeletal Health and Wiser Health Care Units, School of Public Health and Preventive Medicine Monash University St Kilda Australia; 5 Peninsula Clinical School, Central Clinical School Peninsula Health Monash University Frankston Australia; 6 Turning Point Eastern Health Richmond Australia; 7 Outcome Health Surrey Hills Australia

**Keywords:** data linkage, drug policy, general practice, opioid, primary care

## Abstract

**Background:**

Increasing harms related to prescription opioids over the past decade have led to the introduction of a range of key national and state policy initiatives across Australia. These include introducing a mandatory real-time prescription drug–monitoring program in the state of Victoria from April 2020 and a series of changes to subsidies for opioids on the Pharmaceutical Benefit Scheme from June 2020. Together, these changes aim to influence opioid supply and reduce harms related to prescription opioids, yet few studies have specifically explored how these policies have influenced opioid prescribing and related harms in Australia.

**Objective:**

The aim of this study is to examine the impact of a range of opioid-related policies on hospital admissions and emergency department (ED) presentations in Victoria, Australia. In particular, the study aims to understand the effect of various opioid policies and opioid-prescribing changes on (1) the number and rates of ED presentations and hospital admissions attributed to substance use (ie, opioid and nonopioid related) or mental ill-health (eg, suicide, self-harm, anxiety, and depression), (2) the association between differing opioid dose trajectories and the likelihood of ED presentations and hospital admissions related to substance use and mental ill-health, and (3) whether changes in an individual’s opioid prescribing change the risk related to ED presentations and hospital admissions related to substance use and mental ill-health.

**Methods:**

We will conduct a population-level linked data study. General practice health records obtained from the Population Level Analysis and Reporting platform are linked with person-level data from 3 large hospital networks in Victoria, Australia. Interrupted time series analysis will be used to examine the impact of opioid policies on a range of harms, including the rates of presentations related to substance use (opioid and nonopioid) and mental ill-health among the primary care cohort. Group-based trajectory modeling and a case-crossover design will be used to further explore the impact of changes in opioid dosage and other covariates on opioid and nonopioid poisonings and mental ill-health–related presentations at the patient level.

**Results:**

Given that this paper serves as a protocol, there are currently no results available. The deidentified primary health data were sourced from electronic medical records of approximately 4,717,000 patients from 542 consenting general practices over a 6-year period (2017-2022). The submission of results for publication is planned for early 2024.

**Conclusions:**

This study will add to the limited evidence base to help understand the impact of opioid policies in Australia, including whether intended or unintended outcomes are occurring as a result.

**Trial Registration:**

EU PAS Register EUPAS104005; https://www.encepp.eu/encepp/viewResource.htm?id=104006

**International Registered Report Identifier (IRRID):**

DERR1-10.2196/51825

## Introduction

Over the past decade, Australia has witnessed an almost 2-fold increase in opioid-related deaths [1], which led to the introduction of a range of national and state policy changes [2-4]. On April 1, 2020, a mandatory real-time prescription drug–monitoring program (PDMP) was introduced in the state of Victoria [5], which was followed by other Australian jurisdictions also implementing voluntary and mandatory PDMPs [6]. In June 2020, a series of changes to subsidies for opioids on the pharmaceutical benefits scheme were also implemented, including reduced pack sizes, limits on long-term use of opioids for chronic pain, and more restricted indications for opioids [7]. Together, these changes aim to influence opioid supply and reduce harms related to prescription opioids, yet few studies have specifically explored the outcomes of these policy changes in Australia.

In the United States, supply-side policies, including the implementation of PDMPs, were identified as a key precipitating factor for the current opioid crisis, including driving harms related to illicit drug use [8]. In Australia, most opioids are prescribed in primary care settings, and most opioid mortality is still attributed to prescription opioid use [9-11]. Yet, we have little understanding of how similar supply-side interventions are influencing opioid prescribing in primary care and whether changes in prescribing lead to their intended outcome of reducing harms. This is particularly important to study among subgroups of people who are prescribed opioids, such as patients receiving high doses or high-risk medication combinations, who are often the intended target of these policy changes.

Opioid deprescribing has been identified as a key clinical strategy for reducing opioid-related adverse effects and improving quality of life [12-14]. However, policies that have aimed to reduce opioid prescribing have had mixed outcomes elsewhere, including a range of unintended adverse consequences [15,16]. For example, in the United States, hydrocodone rescheduling led to an increase in total opioid prescribing [17], while PDMP implementation led to an increase in heroin-related mortality [15]. Other adverse unintended consequences that have been reported in patients prescribed opioids following opioid restrictions include increases in withdrawal symptoms (eg, uncontrolled pain and psychological distress), uncontrolled pain, psychological distress, illicit opioid use, and suicide attempts [18-20].

Emergency department (ED) presentations and hospital admissions are considered crucial indicators when evaluating outcomes of policies targeting opioid prescribing due to their ability to capture acute incidents (eg, overdose poisonings or opioid-related injuries or accidents), assess the severity of complications, and provide valuable population-level insights [21,22]. Hospitalizations and ED presentations were common outcomes examined in US studies of PDMP implementation [23-25]. Yet, to date, few studies outside the United States have examined similar outcomes following the introduction of opioid-related policies. Given the adverse outcomes reported following the introduction of opioid-prescribing interventions elsewhere, this study seeks to examine the impact of introducing a range of opioid-related policies on hospital admissions and ED presentations in Victoria, Australia. In particular, the study aims to understand the effect of various opioid policy and opioid-prescribing changes on the following:

- The number and rates of ED presentations and hospital admissions attributed to substance use (ie, opioid and nonopioid related) or mental ill-health (eg, suicide, self-harm, anxiety, and depression).
- The association between differing opioid dose trajectories and the likelihood of ED presentations and hospital admissions related to substance use and mental ill-health.
- Whether changes in an individual’s opioid prescribing change the risk related to ED presentations and hospital admissions related to substance use and mental ill-health, after considering other confounding factors, using a case-control design.

## Methods

### Study Design and Setting

This is a retrospective data linkage study using data from Victoria, Australia. Patient-level data from primary health networks (PHNs), extracted from the Population Level Analysis and Reporting (POLAR) platform provided by Outcome Health, and admission and ED data sets from 3 metropolitan hospital networks will be linked. These data were chosen because they provide patient-level demographic, diagnosis, medication, and hospital attendance information, and they can be accessed in a timely manner to address the research questions.

The state of Victoria is the second-most populous state in Australia, with 26% of the nation’s population residing there. The current POLAR database covers approximately 30% of general practices across southeastern Victoria [26]. A total of 3 hospital networks in Victoria (Monash Health, Eastern Health, and Peninsula Health) that align with the Eastern and Southeast PHN catchments were chosen to examine opioid harms due to the high number of POLAR-participating practices in these 3 health regions. These hospital networks are located in the greater Melbourne metropolitan area and have catchments of nearly 2.6 million people, covering 40% of Victoria’s population, with their EDs managing around 500,000 presentations per year. The areas represented by these data are geographically, demographically, and socioeconomically diverse [27].

### Population

The study population will include people 18 years or older who had at least 1 attendance at general practice sites participating in POLAR data provision during the study period (2017-2022).

### Medications of Interest

We will include all opioids prescribed for pain (ie, codeine, dextropropoxyphene, fentanyl, hydromorphone, morphine, oxycodone, pethidine, tapentadol, tramadol, buprenorphine [transdermal products only], and methadone [tablet formulations only]). Formulations that are only used as cough suppressants (eg, dihydrocodeine) or for opioid replacement therapy (eg, high-dose sublingual buprenorphine-naloxone and methadone liquid) will be excluded (Table S1 in Multimedia Appendix 1). The use of nonopioid medicines (eg, gabapentinoids and benzodiazepines) will be considered as covariates.

### Outcomes of Interest

The main outcomes of interest are ED presentations and hospital admissions attributed to substance use (both opioid and nonopioid) or mental ill-health (eg, suicide, self-harm, anxiety, and depression). We focus on these outcomes as opioid- and nonopioid-related substance use, mental health crises, and suicide have all been demonstrated to be key outcomes related to changes in opioid prescribing [18-20].

Primary and secondary diagnoses related to ED presentations and hospital attendance are coded according to the International Statistical Classification of Diseases and Related Health Problems, Tenth Revision, Australian Modification (ICD-10-AM). Opioid-related presentations will be identified where the primary or additional diagnoses include (1) F11.0-F11.9 (opioid-related disorders) and (2) T40.0-T40.4, T40.6 (poisoning by, adverse effects of, and underdosing of narcotics and psychodysleptics).

Non–opioid-related substance use will include substances such as alcohol, heroin, and cannabis. The ICD-10-AM codes F10, F12-F19 will be used to identify cases of unintentional poisoning. Cases of suicide attempts and self-harm that satisfy the following criteria will be included: (1) a principal diagnosis in the ICD-10-AM range S00-T75, T79 (injury, poisoning, and certain other consequences of external causes); (2) the first reported external cause code in the record in the ICD-10-AM range X60-X84, Y87.0 (external causes of morbidity); and (3) suicide-related behaviors (R45.81 [suicidal ideation]). A full list of ICD-10-AM diagnosis codes used to define mental ill-health–related hospital separations by the Australian Institute of Health and Welfare is provided in Table S2 in Multimedia Appendix 1.

### Data Source, Data Variables, and Data Linkage

#### Population-Level Analysis and Reporting Data

Primary health data are collected from constituent general practice clinics in 3 participating Victorian PHN regions (Eastern Melbourne PHN, Gippsland PHN, and Southeastern Melbourne PHN) by the POLAR system. POLAR is a cloud-based clinical intelligence platform that extracts, processes, and organizes deidentified information from clinical information systems within participating practices. The subset of the deidentified primary health data was sourced from electronic medical records of approximately 4,717,000 patients from 542 consenting general practices over a 6-year period (2017-2022). A total of 4 sub–data sets from POLAR will be used for this study:

- Patient data set: It contains data relating to patients’ demographic details such as age, gender, postcode, concession, and marital status.
- Prescription data set: It contains data on medications prescribed by the general practitioner (GP), including the date each prescription was written, generic ingredient name and brand prescribed, medication dose, strength and frequency, and Anatomical Therapeutic Chemical Classification.
- Diagnosis data set: It contains data on patients’ diagnoses as extracted from the clinical information system, including diagnosis recorded dates and documented diagnoses that have been coded and validated by Outcome Health using SNOMED CT (Systematized Nomenclature of Medicine Clinical Terms).
- Activity data set: Details of patient activity at the primary care practices are coded in POLAR, including the type of consultation provided (eg, face-to-face, telehealth, and telephone) and the date the activity occurred.

#### Hospital Data

Monash Health, Eastern Health, and Peninsula Health will provide data on (1) hospital admissions and (2) ED presentations. Hospital admission data contain hospital admission records of admitted patients between 2017 and 2022, including dates of admission, separation date and mode (the process by which an episode of care for an admitted patient ceases), and diagnostic and procedure ICD-10-AM codes. ED data are provided separately with a single record for each presentation episode, including dates of presentation and departure, referral source, arrival mode, visit type, triage, and diagnosis.

#### Data Linkage

POLAR and hospital data will be linked at the patient level. A privacy-preserving method using a set of 3 statistical linkage keys (SHA-256 HASH keys) using personal identifying information contained in each of the data sets is used for data linkage. These linkage keys enable the identification of the same patients at different practices (removing patient duplication) without the need to export personal identifying data. The keys are developed through “ORCA,” specialist linkage software developed and used by Outcome Health that can generate the same linkage keys in each of the hospital systems and the POLAR database.

The linkage keys are generated within the practice and hospital data environments based on available identifiable data fragments (eg, name, gender, date of birth, and Medicare number), and the ORCA tool ensures that identifiable information does not leave the clinical practice environment. Each linkage key is hashed and irreversible; that is, it cannot be used by the researcher to reidentify individuals or their data.

This linkage process follows a method described in detail elsewhere [28]. When the linkage is complete, the linkage keys allow the matching of records across both the hospital and POLAR data. The use of multiple keys and probabilistic matching maximizes linkage accuracy.

Deidentified data with linkage keys will be released by each of the organizations into Monash University’s Secure eResearch Platform, a secure environment that provides a high-powered computing environment that ensures secure storage of linked data with remote access and a clear audit trail of access.

### Covariates

Analyses will adjust for key covariates associated with opioid-related outcomes such as age, sex, physical and mental health comorbidity, and substance use (including alcohol and nicotine use; Textbox 1) [29-34].

**Covariates from the Population Level Analysis and Reporting database.**

- **Sociodemographic**

Age, sex, Socioeconomic Indexes for Areas status for residence, and concessional status

- **Prescription medicine use**

All prescription medicine use and analysis of medication classes of interest including benzodiazepines, gabapentinoids, antidepressants, and antipsychotics

- **Physical health comorbidity**

Documented diagnoses

- **Mental health**

Treatment for mental disorders with medicines, mental health care plans, and documented diagnoses

- **Pain**

Relevant documented diagnoses

#### Mental Health

Individuals will be classified as having a mental health condition if there is evidence of a recorded diagnosis based on the lists of SNOMED (Systematized Nomenclature of Medicine) codes (Table S3 in Multimedia Appendix 1), a prescription for medication used in the treatment of mental health issues, or enrollment in mental health care plans. This integrated approach overcomes a common limitation where some individuals may receive treatment with medication for a mental health condition or a formal diagnosis but not both [35,36]. Mental health conditions will be further categorized into major groups, including anxiety, depressive disorder, psychotic disorder, and suicidal and distressed behaviors.

#### Pain

We will use established lists of SNOMED codes and relevant keywords from previous research (Table S4 in Multimedia Appendix 1) to identify patients diagnosed with pain conditions that are likely to be treated with opioid analgesics [37,38]. However, conditions that are not primarily treated with opioids despite having potentially painful symptoms (eg, epilepsy and seizures) will not be included in our analysis.

#### Comorbidity

A modified Cambridge Multimorbidity Score (CMS) will be used to categorize comorbidities using SNOMED codes in the POLAR data [39]. There have been a number of approaches to measuring comorbidity, with the modified CMS recently validated for use in primary care, offering an advantage over the Cambridge Comorbidity Index, which, while widely used, was developed, and valued for use in secondary hospital settings.

#### Other Prescribed Medicine Use

Other medications of interest that may be prescribed for pain management, including nonsteroidal anti-inflammatory drugs, benzodiazepines, gabapentinoids, and tricyclic antidepressants, will also be extracted.

### Data Preparation and Quality Assessment

Before the analysis, data will be prepared, and a quality assessment undertaken. Preparation involves (1) archiving a copy of primary data on the secure environment in original format and creating a copy for use in further analysis; (2) converting data from the received format (often .csv or .xls format) to formats used in statistical packages such as STATA (StataCorp), R (R Foundation for Statistical Computing), and SPSS (IBM Corp); (3) checking naming conventions for indicators and fields to ensure that these are consistent with data dictionaries; and (4) developing a study data dictionary and creating metadata.

Data validity and quality assurance will be conducted on the linked database. This will involve examining the missingness of individual data fields, inconsistency between data sets, inaccurate and unreasonable data values (eg, prescription quantity >200), identification and removal of duplicate records, and merging multiple episodes that occur within a single presentation (eg, where a patient may move between wards within a hospital).

### Data Exploration and Description

Data exploration and visualization will be undertaken to enable the research team to examine the properties of the data sets, such as dispersion or distribution of values, correlation between variables, and time trends. Descriptive statistics (counts, means, medians, etc) will be calculated based on demographic data, diagnosed conditions, and medication use. Preliminary methods for defining key variables will be developed. Next, we will check the internal consistency of the linked data. This will help quantify the success of data linkage across POLAR, hospital, and ED data. We will define and examine the consistency of a range of indicators, such as patients’ age and gender.

### Analysis Plan

#### Aim 1

The first aim was to examine the effect of opioid policies on the number and rates of ED presentations and hospital admissions attributed to substance use (ie, opioid and nonopioid related) and mental ill-health (eg, suicide, self-harm, anxiety, and depression).

##### Hypotheses

The 3 hypotheses were:

- Hypothesis 1: Reduced prescription opioid harm will follow each policy change.
- Hypothesis 2: Unintended outcomes in the form of non–opioid-related presentations and mental ill-health–related presentations will occur following policy changes restricting opioid access.
- Hypothesis 3: There will be disparities in policy impacts in different populations, with greater reductions in those prescribed opioids and no impact on harm among subpopulations who are not targets of the policy changes (eg, those with cancer pain).

**Figure 1:**
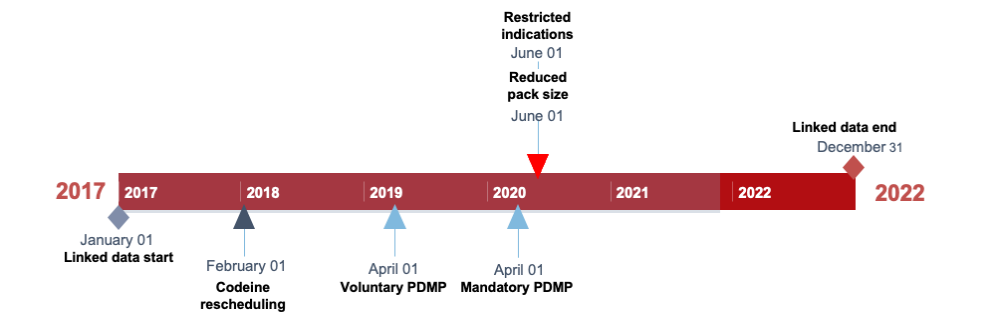
Key exposure of interest. Australian national and state policies relating to opioids. PDMP: prescription drug–monitoring program.

The key policies of interest in this study will be national and state policies that aim to restrict opioid supply (Figure 1).

##### State-Level Policy

Victoria’s PDMP “SafeScript” was implemented in April 2019, before use became mandatory for all community prescribers and pharmacists on April 1, 2020. It indicates potential risk to prescribers and dispensers at the time of medication supply by issuing amber and red-flag alerts, for example, with higher opioid doses (above 50 mg oral morphine equivalent) and high-risk drug combinations such as fentanyl and benzodiazepines [6].

##### National-Level Policy

On February 1, 2018, all codeine preparations became available only on prescription after lower-dose (up to 15 mg) codeine products were removed from “pharmacist only” (Schedule 3) sale. On June 1, 2020, changes were made to the listings of many opioids and multiple formulations listed on the Pharmaceutical Benefits Scheme (PBS) General Schedule. Changes included new listings for smaller pack sizes, increased restrictions on indications, and additional requirements for a second opinion to continue long-term opioid prescribing.

We will use an interrupted time series study design to understand the effects of these policy interventions. This method is widely used in public health research studies, particularly where randomization is neither feasible nor ethical [40]. Interrupted time series goes beyond a simple prepost design by considering the comparison of slopes generated across multiple time points and taking into account the underlying secular trends and random fluctuations. It allows researchers to examine the changes in the outcome variable and determine whether they are attributed to the intervention or other factors.

The primary outcomes of interest for this analysis are changes in ED and hospital admissions related to substance use (opioid and nonopioid) and mental ill-health. A total of 2 types of denominators will be used to estimate monthly rates: total patients’ exposure to opioid prescription in the POLAR primary cohort before and after the policy changes and total patients who had GP consultations in the whole POLAR primary care cohort in the same month.

To evaluate the impact of codeine rescheduling on the same outcomes, the observation period will be 12 months before and 12 months after the policy change, with data points defined by calendar month. The same observation period will be applied to evaluate the impact of the mandated implementation of PDMP, and reduced pack size policies using data from 24 months before and after the intervention change. As these 2 policies were implemented within a 2-month period, we will consider a policy intervention window between April 1, 2020, and June 1, 2020, with outcome data during the intervention implementation period excluded.

Outcomes will be measured by comparing level changes and trends over time during the study period using segmented regression modeling. This method involves partitioning outcome data into time intervals consistent with the times at which intervention interruptions occur and fitting a separate regression line segment to each time interval. Sensitivity analyses using different lags will be conducted to account for autocorrelation and determine the robustness of the primary results. If statistically significant, harmonic terms will be added to the models to account for seasonality. Additional segmented regression models will be used to examine the outcome among certain subpopulations such as patients with cancer diagnoses, those prescribed opioids, and those not prescribed opioids.

#### Aim 2

The second aim was to examine the association between differing opioid dose trajectories and the likelihood of ED presentations and hospital admissions related to substance use (opioid and nonopioid related) and mental ill-health (eg, suicide, self-harm, anxiety, and depression).

##### Hypothesis

Compared with patients with gradual dose discontinuation or a stable opioid dose, those with a rapid dose reduction or a gradual dose increase will have a higher risk for substance use and mental ill-health–related presentations.

This analysis will compare the trajectories of prescription opioid doses and the risk of ED presentations due to opioid-related and other adverse events. This analysis will be limited to patients receiving long-term opioid therapy, defined as those who were prescribed opioids regularly and continuously for 90 days or more [41,42]. Patients with a cancer diagnosis record in the POLAR data will be excluded from this analysis. The exposure of interest in this study is a distinct trajectory of opioid prescriptions among long-term opioid users. We will identify these trajectories by calculating the average daily oral morphine equivalent during the 6 months after the first eligible opioid prescription, when the persistence was identified (ie, 90 days of persistent opioid use and an additional 90 days). The index date is the first day following this 180-day period of long-term opioid therapy. Then, we will identify distinct opioid prescription patterns based on doses over time using group-based trajectory models. This model is a statistical method used to identify subgroups of individuals with similar patterns. The primary outcome is defined as the time to the first ED presentation, captured in the hospital data in the 6 months following the 6-month opioid prescription trajectory measurement period. The Cox proportional hazards models will be used to compare time-to-event, adjusting for covariates. These covariates included demographics (eg, age, gender, and socioeconomic status), characteristics of opioids prescribed, chronic pain diagnoses recorded (eg, musculoskeletal, neuropathic, migraine, and headache), polypharmacy (defined as having 5 or more different medications daily [43]), comorbidities (as measured by the modified CMS [39]), and ED presentations during the trajectory measurement period.

#### Aim 3

The third aim was to determine whether an individual’s opioid-prescribing changes lead to ED presentations related to substance use (opioid and nonopioid related) and mental ill-health (eg, suicide, self-harm, anxiety, and depression), after considering other key covariates using a case-crossover design.

##### Hypothesis

Opioid dose tapering or abrupt discontinuation during the case period will be associated with an increased risk of opioid- and nonopioid-related harms and mental ill-health–related presentations.

This analysis will evaluate whether changes in opioid prescribing increase the risk of related outcomes through the application of a case-crossover design. The case-crossover design is an epidemiological design used for studying potential causes of sudden events and has previously been used for investigating the association between substance use and suicidal behavior [44]. It uses each case as its own control, thereby eliminating the time-invariant characteristics among individuals (eg, age, sex, and chronic substance use). In this study, adverse outcomes will be ED presentations across the 3 hospital data sets. The index date will be defined as the date of arrival. Each enrolled case for the case-crossover analysis will be restricted to patients who received opioids at least once during the hazard or control periods. The hazard period will be 60 days before the index date, and a single hazard period will be matched with 5 sets of 60-day control periods before and after without an ED attendance.

Comorbid mental health conditions will be the main confounding factors and will be assessed during the 12 months before the index date. Individuals will be identified as having a mental health condition using the aforementioned definition. It is common for individuals who die by suicide or attempt suicide to also have additional comorbidities related to mental health disorders. Therefore, referring to previous patient diagnoses, CMS will also be determined for the 12 months before the index date. Concurrent use of benzodiazepines and antidepressants, tricyclic antidepressants, and pregabalin will also be included as a confounding factor. Discordant pairs of these confounders between the hazard and control periods will be adjusted in the outcome models.

Odds ratios and 95% CIs will be determined by conditional logistic regression. We will evaluate the risk of substance use–related attendance for opioid dosage tapering and abrupt discontinuation, adjusting for confounding factors, and perform sensitivity analyses by varying the time window of the exposure period from 60 to 30 days to evaluate the robustness of the results. Additional sensitivity analyses will be performed by varying the number of controls from 5 sets of control periods (main analyses) to 2 or 3 sets of control periods to confirm whether the exposure trend affects the estimated odds ratio.

### Management of Missing Data

To ensure comprehensive and accurate demographic information, a cross-check will be conducted to address missing data in both hospital records and the POLAR data set. When key demographic information is absent in hospital data, the gaps will be filled by leveraging the variable field from the POLAR data. Similarly, in instances where the POLAR data lack essential demographic details, an imputation process will be used based on the available hospital data. In cases where a discrepancy in demographic information is identified between the hospital data and the POLAR data set, priority is given to the values recorded in the hospital records. The demographic details present in the hospital data are considered more reliable and representative of the specific patient population being studied. In addition, where key prescription-related information in the POLAR data is missing (eg, quantity prescribed or dose), these will be imputed based on standard pack sizes and the most common formulations used. Missing data for all variables will be checked and quantified. Multiple imputation methods will be considered where there are more than 5% missing data within variables.

### Ethics Approval

The process of collection, transfer, and storage of general practice data on the POLAR platform was granted ethics approval in August 2017 (NREEC RACGP Protocol ID: 17-008). Consent for the use of deidentified patient data for research extracted and processed on the POLAR platform occurs as follows: practices that contribute data to the POLAR system enter into an agreement with their local PHN. The agreement articulates the responsibility of practices to inform their patients that data are being collected and of their potential use for research. As an additional layer of assurance, practices contributing data to the POLAR platform adhere to the RACGP Accreditation Standards, which stipulate requirements for responsible management of health data. Patients are informed at the point of collection and are presented with the opportunity to opt out. POLAR data are deidentified at the point of collection, according to Outcome Health’s Data Deidentification Decision-Making Guide, developed with guidance from the Office of the Australian Information Commissioner. Hospital data are proposed to be used without the consent of individuals. A multisite ethics application was approved by Monash Health as the lead Human Research Ethics Committee (ID 76744), with site-specific applications approved by Monash Health (RES-22-0000-026A), Peninsula Health (SSA/76744/PH-2022), and Eastern Health (S22-032-76744). A waiver of consent was granted by the respective Human Research Ethics Committees for linkages between the POLAR data and hospital data. Privacy-preserving encrypted linkage software was used to link the data, and deidentified content data were submitted into a secure e-research environment by each of the data custodians for analysis by the research team. Ethics approval has been obtained from the Monash University Human Research Ethics Committee (ID 76744), Monash Health (RES-22-0000-026A), Peninsula Health (SSA/76744/PH-2022), and Eastern Health (S22-032-76744).

## Results

Data linkage commenced in January 2023 and was completed in August 2023. Data analysis has not yet commenced. Submission of results for publication is planned for early 2024.

## Discussion

### Overview

Policies restricting access to medications such as opioids are commonly implemented, with limited evidence of their effect on reducing opioid-related or other harms. This study will add to the limited evidence base to help understand the impact of these policies in Australia, including whether intended or unintended outcomes are occurring as a result. By closely monitoring and analyzing ED presentations and hospital admissions as markers, policy makers can assess the impact of interventions, identify areas for improvement, and drive evidence-based policy changes that effectively address the complex challenges associated with opioid use and promote healthier communities.

### Strengths

This study will link primary care and hospital data to investigate opioid exposure and adverse outcomes. POLAR data contain rich patient-level data, including diagnoses and comorbidities, that are often missing from existing administrative data such as Medicare Benefits Schedule and PBS data in Australia. The linked data set will also capture both publicly subsidized and nonsubsidized prescriptions, making it distinct from the more commonly used PBS (government subsidy) database. Moreover, the linkage key is consistent throughout POLAR participating practices, which will allow for a more comprehensive capture of covariates, particularly coprescribing and hospital readmission.

### Limitations

There are several limitations to note. First, the use of prescribing data does not enable confirmation that prescribed medicines were dispensed or taken by the patient. Second, as both primary care and hospital data are drawn from 3 specific geographical areas, there is potential for selection bias. This concern is lessened by the inclusion of 3 PHNs whose average opioid-prescribing patterns are consistent with national opioid-prescribing patterns [45]. Further, there is established geographic and sociodemographic diversity across these health areas that limits concerns about generalizability [46]. Third, patients who visited non-POLAR GP practices and were admitted to hospitals other than the selected ones are not captured in this study; however, it is noted that the 3 large hospital networks serve the majority of the population in each of the catchment areas. Fourth, a list of ICD-10 codes will be used to identify study outcomes. However, there are limits to the completeness of the coding. For instance, suicide in EDs is commonly recorded using a single diagnostic code [47], and opioid-poisoning presentations can be coded under the more generic code T50: “Poisoning by diuretics and other and unspecified drugs, medicaments and biological substances” [48]. Therefore, our opioid-related poisoning and suicide and self-harm estimates are likely to be an underestimate of the true prevalence. In addition, deaths that occur before reaching the hospital and harm that occur outside the hospital setting (eg, ambulance attendances) would not be captured unless the patient was later transferred to a hospital.

## Appendix

Multimedia Appendix 1 Supplemental materials.

Multimedia Appendix 2 National Health and Medical Research Council (NHMRC, Australia) application assessment summary.

Multimedia Appendix 3 Grant outcome and scoring from the National Health and Medical Research Council (NHMRC, Australia).

## Abbreviations

CMS: Cambridge Multimorbidity Score
ED: emergency department
GP: general practitioner
ICD-10-AM: International Statistical Classification of Diseases and Related Health Problems, Tenth Revision, Australian Modification
PBS: Pharmaceutical Benefit Scheme
PDMP: prescription drug–monitoring program
PHN: primary health network
POLAR: Population Level Analysis and Reporting
SNOMED: Systematized Nomenclature of Medicine Clinical Terms
SNOMED CT: Systematized Nomenclature of Medicine Clinical Terms

## Data Availability

Data sharing is not applicable to this protocol paper as no data sets were generated or analyzed during its writing. Those wishing to access the data should approach Outcome Health (the data custodians of Population Level Analysis and Reporting platform), Monash Health, Eastern Health, and Peninsula Health. Access fees for data and analyses may apply.
