# Supplementary material for: Determining the Impact of Opioid Policy on Substance Use and Mental Health–Related Harms: Protocol for a Data Linkage Study
Source: JMIR Res Protoc. 2023 Oct 17;12:e51825. doi: 10.2196/51825 (PMC10618880; doi:10.2196/51825)
Supplement: Multimedia Appendix 1 [file resprot_v12i1e51825_app1.docx]

**Table S1**. Eligibility criteria for the cohort.

| **Medications** | Our cohort was defined based on their use of the following opioids: | |
| --- | --- | --- |
|  | **Opioid** | **ATC Codes** |
|  | Buprenorphine | N02AE01 |
|  | Codeine and codeine combinations | R05DA04, N02AJ06, N02AJ07,N02AJ08  N02AJ09 |
|  | Fentanyl | N02AB03 |
|  | Hydromorphone | N02AA03 |
|  | Methadone | N02AC52 |
|  | Morphine | N02AA01 |
|  | Oxycodone | N02AA05 |
|  | Oxycodone-naloxone | N02AA55 |
|  | Tramadol and tramadol combinations | N02AX02  N02AJ13 |
|  | Tapentadol | N02AX06 |
|  | Pethidine | N02AB02 |
|  | Dextropropoxyphene | N02AC04 |

| ICD-10 Codes | Description |
| --- | --- |
| F00-F09 | Organic, including symptomatic, mental disorders |
| F20‐F29 | Schizophrenia, schizotypal and delusional disorders |
| F30‐F39 | Mood (affective) disorders |
| F40‐F48 | Neurotic, stress‐related and somatoform disorders |
| F50‐F59 | Behavioural syndromes associated with physiological disturbances and physical factors |
| F60‐F69 | Disorders of adult personality and behaviour |
| F80‐F89 | Disorders of psychological development |
| F99 | Unspecified mental disorder |
| G47 | Sleep disorders |
| O99.3 | Mental disorder nervous system pregnancy and birth |
| R44 | Other symptoms and signs involving general sensations and perceptions |
| R45.0 | Nervousness |
| R45.1 | Restlessness and agitation |
| R45.4 | Irritability and anger |
| Z00.4 | General psychiatric examination, not elsewhere classified |
| Z03.2 | Observation for suspected mental and behavioural disorder |
| Z04.6 | General psychiatric examination, requested by authority |
| Z13.3 | Special screening examination for mental and behavioural disorders |
| Z50.2 | Alcohol rehabilitation |
| Z50.3 | Drug rehabilitation |
| Z63.1 | Problems relationship w parents & in-laws |
| Z63.8 | Other spec problems related to prim support group |
| Z63.9 | Problem related to primary support group, unspecified |
| Z65.8 | Other specified problems related to psychosocial circumstances |
| Z65.9 | Problem related to unspecified psychosocial circumstances |
| Z71.4 | Counselling and surveillance for alcohol use disorder |
| Z71.5 | Counselling and surveillance for drug use disorder |

**Table S2**. ICD-10-AM diagnosis codes used to define mental health-related hospital separations.

**Table S3**. SNOMED codes used to define mental health-related disorders.

| **SNOMED Code** | **SNOMED Text** | **SNOMED Code** | **SNOMED Text** |
| --- | --- | --- | --- |
| 35489007 | Depression | 44376007 | Dissociative disorder |
| 231504006 | Mixed anxiety and depressive disorder | 17155009 | Trichotillomania |
| 58703003 | Postpartum depression | 231496004 | Hypomania |
| 87414006 | Reactive depression (situational) | 274952002 | Borderline schizophrenia |
| 370143000 | Major depressive disorder | 35919005 | Pervasive developmental disorder |
| 300706003 | Endogenous depression | 260994008 | Bipolar |
| 78667006 | Dysthymia | 568005 | Tic disorder |
| 191616006 | Recurrent depression | 83482000 | Body dysmorphic disorder |
| 73867007 | Severe major depression with psychotic features | 397923000 | Somatisation disorder |
| 310495003 | Mild depression | 76105009 | Cyclothymia |
| 310497006 | Severe depression | 74142004 | Feeding disorder of infancy OR early childhood |
| 192080009 | Chronic Depression | 191714002 | Dissociative convulsions |
| 247803002 | Seasonal affective disorder | 13601005 | Paranoid personality disorder |
| 83458005 | Agitated depression | 26665006 | Antisocial personality disorder |
| 7866700 | Dysthymia | 430909002 | Conduct disorder |
| 192079006 | Postviral depression | 66347000 | Impulse control disorder |
| 366979004 | Depressed mood | 18193002 | Hypochondriasis |
| 197480006 | Anxiety disorder | 473456000 | Compulsive personality disorder |
| 21897009 | Generalised anxiety disorder | 439960005 | Binge eating disorder |
| 47505003 | Post-traumatic stress disorder | 31611000 | Multiple personality disorder |
| 371631005 | Panic disorder | 129606007 | Frontal lobe syndrome |
| 191736004 | Obsessive-compulsive disorder | 191667009 | paranoid disorder |
| 25501002 | Social phobia | 87991007 | Gender identity disorder |
| 70691001 | Agoraphobia | 52954000 | Schizoid personality disorder |
| 11806006 | Separation anxiety disorder of childhood | 193756007 | Charles Bonnet syndrome |
| 386810004 | Phobic disorder | 80711002 | Narcissistic personality disorder |
| 58535001 | Physical & emotional exhaustion state | 31027006 | Schizotypal personality disorder |
| 426174008 | Chronic stress disorder | 111475002 | Neurosis |
| 67195008 | Acute stress disorder | 26472000 | Paraphrenia |
| 10586006 | Occupation-related stress disorder | 69361009 | Kleptomania |
| 231528008 | Anxious personality disorder | 21586000 | Munchausen's syndrome |
| 48694002 | Anxiety | 3298001 | Amnestic disorder |
| 225624000 | Panic attack | 33449004 | Personality disorder |
| 247853008 | Fear of flying | 20010003 | Borderline personality disorder |
| 300895004 | Anxiety attack | 72366004 | Eating disorder |
| 207363009 | Anxiety neurosis | 78004001 | Bulimia nervosa |
| 386808001 | Phobia | 2776000 | Delirium |
| 19887002 | Claustrophobia | 48500005 | delusional disorder |
| 279622009 | Performance anxiety | 74732009 | Mental disorder |
| 126943008 | Separation anxiety | 2073000 | Delusions |
| 247854002 | Flying phobia | 225452001 | Paranoid delusion |
| 191733007 | Fear of pregnancy | 417233008 | Paranoid ideation |
| 191708009 | Chronic anxiety | 216004 | Delusion of persecution |
| 34563004 | Fear of getting cancer | 56882008 | Anorexia nervosa |
| 231501003 | Needle phobia | 46206005 | Mood disorder |
| 247808006 | Anxiety about body function or health | 406506008 | Attention deficit hyperactivity disorder |
| 70997004 | Mild anxiety | 225658008 | Mood stable |
| 102931001 | Fear of heart attack | 79015004 | Worried |
| 79823003 | Panic | 280427006 | Psychotic symptom present |
| 38617005 | Dental phobia | 277843001 | Behavioural problem |
| 102927007 | Nyctophobia | 160822004 | Relationship problems |
| 191709001 | Recurrent anxiety | 75408008 | Feeling angry |
| 191526005 | Schizophrenic disorders | 24199005 | Feeling agitated |
| 18260003 | Postpartum psychosis | 284504009 | Difficult to manage behaviour |
| 38368003 | Schizoaffective disorder, bipolar type | 230736007 | Transient global amnesia |
| 69482004 | Korsakoff's psychosis | 596004 | Premenstrual dysphoric disorder |
| 441704009 | Affective psychosis | 55929007 | Feeling irritable |
| 58214004 | Schizophrenia | 61372001 | Aggression |
| 69322001 | Psychotic disorder | 224965009 | Grief finding |
| 68890003 | Schizoaffective disorder | 18963009 | Mood swings |
| 64905009 | Paranoid schizophrenia | 309838005 | Emotional upset |
| 83746006 | Chronic schizophrenia | 44548000 | Hyperactive behaviour |
| 61831009 | Induced psychotic disorder | 12479006 | compulsive behaviour |
| 191542003 | Catatonic schizophrenia | 112082005 | inappropriate behaviour |
| 31658008 | Chronic paranoid schizophrenia | 422650009 | Social isolation |
| 267073005 | Suicidal | 424196004 | Feeling nervous |
| 6471006 | Suicidal thoughts | 248122005 | Bingeing |
| 82313006 | Suicide attempt | 286647002 | Low self-esteem |
| 248061004 | Self-harm | 90091006 | Breath holding with temper |
| 130968006 | Self-mutilation | 112091009 | Obsessive-compulsive personality trait |
| 69328002 | Distress | 60814007 | Antisocial behaviour |
| 13746004 | Bipolar disorder | 37057007 | Psychophysiologic disorder |
| 260994008 | Bipolar | 28669007 | Anhedonia |
| 371596008 | Bipolar I disorder | 284512001 | Hypomanic behaviour |
| 69328002 | Distress | 248025009 | Hoarding |
| 73595000 | Stress | 263897001 | Feeling tense |
| 102497008 | Symptoms of stress | 66551002 | Psychogenic headache |
| 248265004 | Work stress | 47695004 | inability to cope |
| 276074009 | stress at work | 30819006 | Dysphoric mood |
| 17226007 | Adjustment disorder | 18941000 | Oppositional defiant disorder |
| 57194009 | Adjustment disorder with depressed mood | 231494001 | Mania |
| 47372000 | Adjustment disorder with anxious mood | 5158005 | Gilles de la Tourette's syndrome |
| 162313000 | Morbid jealousy | 18085000 | Compulsive gambling |
| 33323008 | Somatic delusion disorder | 93461009 | Gender dysphoria |
| 191746002 | Psychasthenic neurosis |  |  |
| 238974009 | Delusions of infestation |  |  |

**Table S4**. SNOMED codes used to define pain condition.

| **SNOMED Code** | **SNOMED Text** | **SNOMED Code** | **SNOMED Text** |
| --- | --- | --- | --- |
| 225358003 | Wound care | 421262002 | Tongue swelling |
| 299709002 | Dental abscess | 274172008 | Laceration of finger |
| 309431009 | Arthroscopy of knee | 387801000 | Cervical arthritis |
| 44558001 | Repair of inguinal hernia | 65978000 | Injury of chest wall |
| 47534009 | Decompression of median nerve | 78314001 | Osteogenesis imperfecta |
| 417746004 | Traumatic injury | 263232003 | Fracture of distal end of femur |
| 387731002 | Laminectomy | 74333002 | Spasmodic torticollis |
| 282026002 | Soft tissue injury | 425322008 | Stab wound |
| 300025007 | Excision of basal cell carcinoma | 47693006 | Rupture of appendix |
| 312608009 | Laceration | 180030006 | Amputation of the foot |
| 213299007 | Postoperative pain | 406444002 | Laryngeal spasm |
| 65801008 | Excision | 37625005 | Repair of entropion |
| 450434000 | Excision of squamous cell carcinoma | 95883001 | Bacterial meningitis |
| 312968005 | Excision biopsy of skin lesion | 84744001 | Repair of hernia of abdominal wall |
| 4365001 | Surgical repair | 451000119106 | Closed injury of head |
| 609588000 | Total knee replacement | 83561009 | Spinal stenosis in cervical region |
| 396275006 | Osteoarthritis | 426284001 | Chemical burn |
| 64859006 | Osteoporosis | 33910007 | Postoperative infection |
| 312894000 | Osteopenia | 6836001 | Injury of brachial plexus |
| 90560007 | Gout | 25236004 | Replacement of tricuspid valve |
| 239873007 | Osteoarthritis of knee | 177222006 | Repair of episiotomy |
| 3723001 | Arthritis | 608787004 | Open reduction of fracture of patella with internal fixation |
| 69896004 | Rheumatoid arthritis | 81498004 | Bursitis of hip |
| 239872002 | Osteoarthritis of hip | 430698003 | Replacement of total knee joint |
| 65323003 | Polymyalgia rheumatica | 195742007 | Acute lower respiratory tract infection |
| 239880009 | Osteoarthritis of lumbar spine | 85624009 | Closed reduction of dislocation of shoulder |
| 161891005 | Backache | 47374004 | Tarsal tunnel syndrome |
| 279039007 | Low back pain | 83876008 | Bursectomy |
| 45326000 | Shoulder pain | 56246009 | Hypertrophy |
| 81680005 | Neck pain | 941000119103 | Strain of knee |
| 23056005 | Sciatica | 239727002 | Rupture of posterior cruciate ligament |
| 29857009 | Chest pain | 298591003 | Scoliosis of lumbar spine |
| 57054005 | Acute myocardial infarction | 73795002 | Acute myocardial infarction of inferior wall |
| 194828000 | Angina | 95655001 | Ophthalmic migraine |
| 401314000 | Acute non-ST segment elevation myocardial infarction | 52329006 | Fracture, open |
| 22298006 | Myocardial infarction | 608785007 | Open reduction of fracture of radius and ulna with internal fixation |
| 102589003 | Atypical chest pain | 407496005 | Infection due to Human parainfluenza virus 1 |
| 82423001 | Chronic pain | 416816003 | Ligamentous strain |
| 134407002 | Chronic back pain | 427191004 | Intermetatarsal bursitis |
| 54150009 | Upper respiratory tract infection | 264569006 | Secondary infection |
| 68566005 | Upper respiratory tract infection | 209353008 | Fracture dislocation or subluxation foot |
| 281794004 | Viral upper respiratory tract infection | 303650005 | Removal of mole of skin by excision |
| 4740000 | Herpes zoster | 441809006 | Osteonecrosis of jaw |
| 40733004 | Infection | 398126006 | Muscular headache |
| 50417007 | Lower respiratory tract infection | 263193000 | Supracondylar fracture of humerus |
| 34014006 | Viral infection | 23036009 | Open reduction of fracture of radius and ulna |
| 197927001 | Recurrent urinary tract infection | 281352004 | Iliopsoas bursitis |
| 312118003 | Bacterial upper respiratory infection | 247369005 | Facet joint pain |
| 76844004 | Wound infection | 20701002 | Open reduction of fracture with internal fixation |
| 108365000 | Infection of skin | 56688005 | Clostridial infection |
| 427898007 | Infection of tooth | 38343000 | Vaginal pain |
| 275498002 | Respiratory tract infection | 63043004 | Injury of artery |
| 307759003 | Helicobacter pylori gastrointestinal tract infection | 737263004 | Crushing injury of vertebral region |
| 128477000 | Abscess | 54398005 | Acute upper respiratory infection |
| 37796009 | Migraine | 7523003 | Injury of thigh |
| 25064002 | Headache | 65619001 | Rectovaginal fistula |
| 398057008 | Tension-type headache | 239342001 | Excision of the trapezium |
| 4473006 | Migraine with aura | 444158007 | Injury of cruciate ligament of knee |
| 279016001 | Cervicogenic headache | 19824006 | Skin and subcutaneous tissue infection |
| 56097005 | Migraine without aura | 11114002 | Sore gums |
| 431237007 | Chronic headache disorder | 274743004 | Swelling of inguinal region |
| 23186000 | Menstrual migraine | 44364007 | Amputation of thumb |
| 30989003 | Knee pain | 174391002 | Drainage of ischiorectal abscess |
| 125605004 | Fracture of bone | 405754008 | Cervical spinal cord injury |
| 202882003 | Plantar fasciitis | 302941001 | Nonunion of fracture |
| 49218002 | Hip pain | 67878002 | Abscess of upper arm |
| 40799003 | Subacromial bursitis | 20511007 | Fracture of hand |
| 202855006 | Lateral epicondylitis | 3199001 | Sprain of shoulder |
| 7674000 | Trochanteric bursitis | 448589005 | Arthritis of hand |
| 387800004 | Cervical spine Degeneration | 35726004 | Sprain of lateral collateral ligament of knee |
| 4106009 | Rotator cuff syndrome | 263249005 | Fracture of cuboid |
| 47933007 | Foot pain | 17329003 | Ureteric colic |
| 76107001 | Spinal stenosis | 289475007 | Swelling of vulva |
| 55300003 | Muscle cramp | 186659004 | Herpangina |
| 33737001 | Fracture of rib | 67721000 | Sacroiliac arthrodesis |
| 68962001 | Myalgia | 64234005 | Bone structure of patella |
| 77547008 | Degeneration of intervertebral disc | 177281002 | Excision of melanoma |
| 125601008 | Injury of knee | 221695002 | Achilles bursitis |
| 50448004 | Fracture of vertebral column | 442205007 | Stress fracture of tibia |
| 54404000 | Cervical radiculopathy | 217225009 | Repair of shoulder |
| 399114005 | Adhesive capsulitis of shoulder | 285386001 | Right sided chest pain |
| 247373008 | Ankle pain | 1214006 | Infection by Strongyloides |
| 298382003 | Scoliosis deformity of spine | 428638009 | Encephalitis due to Herpesviridae |
| 44465007 | Sprain of ankle | 30292005 | Mononeuritis multiplex |
| 386207004 | Temporomandibular joint-pain-dysfunction syndrome | 111243002 | Bursitis of knee |
| 274142002 | Dupuytren's contracture | 47833006 | Open reduction of fracture of radius |
| 84017003 | Bursitis | 179412008 | Hybrid unicompartmental knee replacement |
| 56608008 | Pain in wrist | 263134008 | Ligament rupture |
| 57676002 | Joint pain | 125319009 | Surgical fistula |
| 125594001 | Injury of shoulder region | 179353004 | Revision uncemented total knee replacement |
| 123798002 | Lumbosacral spondylosis | 321171000119102 | Lumbar spondylolisthesis |
| 48532005 | Muscle strain | 102616008 | Painful mouth |
| 21794005 | Radial styloid tenosynovitis | 275320004 | Injury of nail |
| 397529006 | Finding of tear meniscus | 385942004 | wound care management |
| 5913000 | Fracture of neck of femur | 38964008 | Arthroscopy of knee with lateral meniscectomy |
| 399346004 | Supraspinatus tear | 275393007 | Oral infection |
| 279038004 | Thoracic back pain | 84020006 | Intervertebral disc structure of fourth lumbar vertebra |
| 125603006 | Injury of ankle | 307534009 | Urinary tract infection in pregnancy |
| 57406009 | Carpal tunnel syndrome | 279029001 | Pain in cervical spine |
| 247398009 | Neuropathic pain | 309430005 | Excision of skin tag |
| 22253000 | Pain | 58103005 | Perianal fistula |
| 27355003 | Toothache | 75851004 | Scalp tenderness |
| 162397003 | Pain in throat | 16443000 | Repair of lop ear |
| 125671007 | Rupture | 202336002 | Acetabular labrum tear |
| 278414003 | Pain management | 449619004 | Swelling of upper arm |
| 16001004 | Otalgia | 262921003 | Injury of testis |
| 71307009 | Ganglion cyst | 65061006 | Open reduction of fracture of tibia |
| 263834005 | Partial tear | 392236004 | Operative procedure on spinal structure |
| 53430007 | Pain of breast | 88522004 | Umbilical pain |
| 21522001 | Abdominal pain | 203556007 | Deviation of finger |
| 10743008 | Irritable bowel syndrome | 20781004 | Partial excision of nail and nail matrix |
| 7093002 | Renal colic | 95880003 | Soft tissue infection |
| 79922009 | Epigastric pain | 102614006 | Generalised abdominal pain |
| 33339001 | Psoriasis with arthropathy | 86044005 | Amyotrophic lateral sclerosis |
| 425940002 | Olecranon bursitis | 266463007 | Iliopsoas abscess |
| 16114001 | Fracture of ankle | 31413008 | Operative procedure on coronary artery |
| 45352006 | Spasm | 40095003 | Renal injury |
| 44946007 | Repair of umbilical hernia | 3321001 | Renal abscess |
| 49650001 | Dysuria | 429350001 | Arthropathy of spinal facet joint |
| 21983002 | Hashimoto thyroiditis | 162049009 | Left flank pain |
| 82065001 | Fracture of carpal bone | 237473006 | Rupture of breast implant |
| 262965006 | Strain of back muscle | 263246003 | Fracture of talus |
| 74323005 | Pain in elbow | 186464008 | Mycoplasma infection |
| 22778000 | Venipuncture | 8217007 | Arachnoiditis |
| 52734007 | Total replacement of hip | 5587004 | Corneal dystrophy |
| 105629000 | Chlamydial infection | 249966004 | Spasmodic movement |
| 53057004 | Hand pain | 282780003 | Heel injury |
| 274152003 | Spondylolisthesis | 250564007 | Measurement of partial pressure of carbon dioxide in blood |
| 69031006 | Excision of breast tissue | 33719002 | Subcutaneous rheumatoid nodule |
| 279040009 | Mechanical low back pain | 28466007 | Juvenile osteochondrosis of second metatarsal |
| 125666000 | Burn | 448394006 | Arthritis of foot |
| 276433004 | Insect bite - wound | 111183001 | Abscess of toe |
| 12676007 | Fracture of radius | 43846000 | Sclerosis |
| 82271004 | Head injury | 125622002 | Dislocation of ankle |
| 415692008 | Bunion | 262560006 | Penetrating wound |
| 81102000 | Injury of back | 281450006 | Loosening of total knee replacement |
| 278862001 | Acute low back pain | 58347006 | Excision of lymph node |
| 66308002 | Fracture of humerus | 307225003 | Perianal pain |
| 308492005 | Contusion - lesion | 95675005 | Ulnar neuritis |
| 384709000 | Sprain | 254502002 | Arthroscopy of elbow |
| 55705006 | Spinal arthrodesis | 239329001 | Excision of bone |
| 53286005 | Medial epicondylitis of elbow joint | 225573003 | Nipple painful |
| 102570003 | Inguinal pain | 448804008 | Iliac vein compression syndrome |
| 65124004 | Swelling | 445008009 | Ganglion cyst |
| 33359002 | Degeneration | 430725003 | Patellofemoral stress syndrome |
| 267039000 | Swollen ankle | 239865003 | Osteoarthritis of acromioclavicular joint |
| 125599006 | Injury of hand | 370471003 | Lumbosacral stenosis |
| 125604000 | Injury of foot | 302017001 | Sore eye |
| 72274001 | Nerve root disorder | 431737008 | Acute lower urinary tract infection |
| 52011008 | Injury of finger | 64576003 | Abscess of buttock |
| 281812007 | Arthroscopy of shoulder | 95417003 | Primary fibromyalgia syndrome |
| 50465008 | Hernia repair | 45456005 | Renal infarction |
| 128196005 | Lumbar radiculopathy | 53059001 | Replacement of mitral valve |
| 243338005 | Nerve root compression syndrome | 703945007 | Laparoscopic repair of hernia of anterior abdominal wall |
| 297147009 | Atrophy of vagina | 236501004 | PFR - Pelvic floor repair |
| 260649000 | Lumbar microdiscectomy | 6574001 | Necrosis |
| 387741004 | Excision of cyst of ovary | 128187005 | Vascular headache |
| 48440001 | Articular gout | 239426007 | Repair of anterior cruciate ligament of knee joint |
| 271857006 | Loin pain | 263208005 | Fracture of distal end of radius and ulna |
| 263251009 | Metatarsal bone fracture | 79216009 | Abscess of groin |
| 55260003 | Calcaneal spur | 274166008 | Scalp laceration |
| 39848009 | Whiplash injury to neck | 230570005 | Burning feet syndrome |
| 35646002 | Excision of lesion of skin | 69195002 | Degeneration of cervical intervertebral disc |
| 26036001 | Obstruction | 323311000119107 | Osteoarthritis of right hip joint |
| 102588006 | Chest wall pain | 16726004 | Renal osteodystrophy |
| 60168000 | Osteomyelitis | 276436007 | Hereditary macular dystrophy |
| 278860009 | Chronic lower back pain | 281810004 | Aspiration of knee joint |
| 123971006 | Colles' fracture | 428041004 | Subacute granulomatous thyroiditis |
| 2733002 | Heel pain | 249531009 | Bowel spasm |
| 302932006 | Tear of medial meniscus of knee | 282756002 | Jaw injury |
| 67801009 | Tenosynovitis | 63901009 | Pain in testicle |
| 9631008 | Ankylosing spondylitis | 384643000 | Repair of tricuspid valve |
| 399112009 | Seronegative arthritis | 279079003 | Dysaesthesia |
| 418363000 | Itching of skin | 69916004 | Fracture of base of thumb |
| 17059001 | Prepatellar bursitis | 193039006 | Complicated migraine |
| 65546002 | Extraction of wisdom tooth | 271587009 | Stiffness |
| 42059000 | Retinal detachment | 83132003 | Upper abdominal pain |
| 77493009 | Fracture of pelvis | 284549007 | Laceration of hand |
| 58150001 | Fracture of clavicle | 65759007 | Injury of mouth |
| 239960007 | Impingement syndrome of shoulder region | 249918006 | Shoulder stiff |
| 82127005 | Perianal abscess | 277762005 | Lumbar puncture |
| 297217002 | Rib pain | 202843000 | Full thickness rotator cuff tear |
| 416462003 | Wound | 300885006 | Swelling of breast |
| 55464009 | Systemic lupus erythematosus | 26696002 | Chemical burn |
| 35566002 | Haematoma | 201796004 | Systemic onset juvenile chronic arthritis |
| 56060000 | Repair of musculotendinous cuff of shoulder | 26460006 | Slipped upper femoral epiphysis |
| 239720000 | Tear of meniscus of knee | 49388007 | Sprain of foot |
| 60046008 | Pleural effusion | 287097007 | Sprained finger/thumb |
| 367444000 | Repair of vagina | 8971008 | Psychalgia |
| 62994001 | Tracheitis | 239293007 | Internal fixation of fracture |
| 282770002 | Injury of ribs | 239592001 | Forefoot amputation |
| 252991009 | Cervical intraepithelial neoplasia grade III with severe dysplasia | 112977000 | Repair of diaphragmatic hernia |
| 36071006 | Chondromalacia of patella | 162050009 | Right flank pain |
| 8847002 | Spondylosis | 55130001 | Laryngotracheitis |
| 56717001 | Tuberculosis | 762452003 | Chronic musculoskeletal pain |
| 289900009 | Period pain present | 71772004 | Vasospasm |
| 300954003 | Pain in calf | 239312004 | Osteotomy of proximal tibia |
| 238187004 | Repair of incisional hernia | 4568003 | Retrosternal pain |
| 281255004 | Small bowel obstruction | 197233000 | Perianal infection |
| 67315001 | Osteoarthritis of shoulder | 90834002 | Pain in limb |
| 239431009 | Reconstruction of anterior cruciate ligament of knee joint | 608872001 | Open reduction of dislocation of patellofemoral joint |
| 58718002 | Rheumatic fever | 208388003 | Fracture at wrist and/or hand level |
| 201038005 | Corn - lesion | 279001004 | Pain finding at anatomical site |
| 239725005 | Rupture of anterior cruciate ligament | 1261007 | Fracture of multiple ribs |
| 193031009 | Cluster headache | 267096005 | Frontal headache |
| 35678005 | Multiple joint pain | 274173003 | Laceration of thumb |
| 282776008 | Injury of toe | 71303008 | Atypical facial pain |
| 198130006 | Pelvic inflammatory disease | 441702008 | Strain of muscle and/or tendon of wrist |
| 302490008 | Arthroscopic meniscectomy | 62224002 | Abscess of appendix |
| 285365001 | Pain in toe | 59687004 | Excision of laryngeal polyp |
| 125598003 | Injury of wrist | 162042000 | Abdominal wall pain |
| 26294005 | Radical prostatectomy | 209783002 | Rupture plantaris tendon |
| 392021009 | Lumpectomy of breast | 301013004 | Pseudomonas urinary tract infection |
| 248480008 | Leg swelling symptom | 10000006 | Radiating chest pain |
| 417076003 | Dislocation of shoulder joint | 46866001 | Fracture of lower limb |
| 30085007 | Morton's metatarsalgia | 128526009 | Smooth muscle spasm |
| 81060008 | Intestinal obstruction | 64455005 | Fracture of acetabulum |
| 21351003 | Fracture of phalanx of foot | 302391008 | Curettage and cauterisation of skin lesion |
| 41699000 | Effusion | 171841007 | Endoscopic carpal tunnel release |
| 2089002 | osteitis deformans | 314668006 | wedge fracture of vertebra |
| 78715003 | Subdeltoid bursitis | 84062004 | Juvenile osteochondrosis of carpal lunate |
| 202381003 | Knee joint effusion | 263227004 | Midcervical fracture of neck of femur |
| 247081001 | Posterior vitreous detachment | 372939007 | Suppurative arthritis |
| 401303003 | Acute ST segment elevation myocardial infarction | 400012003 | Abrasion and/or friction burn of skin |
| 119416008 | Epigastric discomfort | 65551008 | Radical retropubic prostatectomy |
| 74400008 | Appendicitis | 60404007 | Cerebral abscess |
| 31975004 | Fracture of scaphoid bone of wrist | 162181003 | Vaginal discomfort |
| 312119006 | Bacterial lower respiratory infection | 14232007 | Cryptococcal meningitis |
| 302399005 | Punch biopsy of skin lesion | 240532009 | Human papillomavirus infection |
| 118442004 | Drainage of abscess | 200833005 | Superficial sunburn |
| 82119001 | Thyroiditis | 111253001 | Infection of bone |
| 6617009 | Referred pain | 77140003 | Superficial burn injury |
| 176761007 | Large loop excision of transformation zone | 268236002 | Congenital bladder neck stenosis |
| 373621006 | Chronic pain syndrome | 289524006 | Swelling of vagina |
| 208393000 | Fracture of metacarpal bone | 15686041000119102 | Osteoarthritis of right temporomandibular joint |
| 91019004 | Paraesthesia | 279062009 | myofascial pain |
| 37389005 | Biliary colic | 441564005 | Stress fracture of calcaneus |
| 300956001 | Low back strain | 263172003 | Fracture of mandible |
| 44323002 | Tuberculosis of kidney | 161972006 | Central chest pain |
| 4386001 | Bronchospasm | 263164000 | Fracture of zygoma |
| 186182003 | Tuberculosis of pleura | 43478001 | Abdominal tenderness |
| 90660004 | Application of dressing for burn | 95340003 | Nail bed infection |
| 41652007 | Pain in eye | 247390002 | Sciatic nerve palsy |
| 25055007 | Ingrowing nail with infection | 102447009 | Postmenopausal osteoporosis |
| 111985007 | Chronic abdominal pain | 24856003 | Diagnostic radiography of coccyx |
| 85848002 | Corneal abrasion | 209635006 | Rupture of lateral ankle ligament |
| 281923005 | Crush fracture of thoracic vertebra | 281535004 | Fracture of lateral malleolus |
| 262520005 | Thumb injury | 403149008 | Spider bite wound |
| 279084009 | Chest discomfort | 17440005 | Anal spasm |
| 428220001 | Tear of skin | 238202001 | Repair of parastomal hernia |
| 4557003 | Preinfarction syndrome | 315248002 | Intractable breast pain |
| 269113006 | acute meniscal tear, medial | 263030002 | Dislocation of toe joint |
| 281933002 | Crush fracture of lumbar vertebra | 263252002 | Fracture of base of fifth metatarsal |
| 83062006 | Infection by Candida albicans | 128069005 | Injury of abdomen |
| 31978002 | Fracture of tibia | 429340002 | Traumatic injury of skeletal muscle |
| 127294003 | Traumatic &/OR non-traumatic brain injury | 309462008 | Infection of big toe |
| 299989006 | Infection toe | 61960001 | Lordosis deformity of spine |
| 263225007 | Fracture of proximal end of femur | 428786006 | Localised superficial swelling of skin |
| 128200000 | Complex regional pain syndrome | 172410004 | Debridement of corneal lesion |
| 125667009 | Contusion | 275247006 | Keller's excision arthroplasty |
| 235395005 | Excision of pilonidal sinus | 19207007 | Manipulation |
| 239961006 | Bursitis of shoulder | 208444006 | closed fracture finger proximal phalanx |
| 79962008 | Diffuse spasm of oesophagus | 277321001 | Phrenic nerve lesion |
| 2237002 | Pleuritic pain | 231794000 | Injury of globe of eye |
| 299991003 | Infection of finger | 230477005 | Chronic post-traumatic headache |
| 69650005 | Arthroscopy of hip | 53801007 | Ganglioneuroma |
| 718854004 | Injection of steroid into trochanteric bursa | 93153005 | Limb-girdle muscular dystrophy |
| 399912005 | Pressure ulcer | 428671008 | Arthropathy of lumbar facet joint |
| 428883008 | Rupture of tendon of biceps | 441806004 | Abscess of brain |
| 135851009 | Hamstring injury | 32021005 | Pulp abscess of finger |
| 263171005 | Fractured nasal bones | 263250005 | Fracture of bone of forefoot |
| 10085004 | Metatarsalgia | 274040007 | Hypospadias repair |
| 371186005 | Amputation of toe | 21092002 | Abscess of finger |
| 312133006 | Viral respiratory infection | 6072800 | Abdominal swelling |
| 240197007 | Stress fracture | 724905004 | Injury of muscle at shoulder level |
| 279043006 | Pain in buttock | 9730000 | Arthrodesis of hip |
| 407450002 | Herpes simplex type 2 infection | 7199000 | Tuberous sclerosis syndrome |
| 398102009 | Acute poliomyelitis | 282028001 | Multiple-resistant Staphylococcus aureus infection |
| 417373000 | Inflammatory polyarthropathy | 445942002 | Excision of temporal lobe |
| 34789001 | Pain in the coccyx | 162011005 | Sore mouth |
| 48210000 | Lumbosacral spondylosis without myelopathy | 87866006 | Trismus |
| 161882006 | Stiff neck | 202478007 | Sternoclavicular joint pain |
| 120053002 | Fallopian tube excision | 446114009 | Excision of carcinoma of nose |
| 429513001 | Rupture of Achilles tendon | 281530009 | Fracture of ulnar styloid |
| 428794004 | Fistula | 287425000 | Palate excision |
| 422588002 | Aspiration pneumonia | 52478002 | Subdiaphragmatic abscess |
| 195708003 | Recurrent upper respiratory tract infection | 176132003 | Repair of rupture of bladder |
| 202487003 | Sacroiliac joint pain | 443786003 | Injury whilst engaged in sports activity |
| 279069000 | Musculoskeletal pain | 239796000 | Juvenile chronic arthritis |
| 407451003 | Herpes simplex type 1 infection | 81902001 | Sprain of medial collateral ligament of knee |
| 239874001 | Osteoarthritis of ankle | 238435009 | Steroid-modified tinea infection |
| 247347003 | Discomfort | 234506007 | Rupture of spleen |
| 51037009 | Fracture of patella | 423561003 | Community-acquired methicillin-resistant Staphylococcus aureus infection |
| 78514002 | Thigh pain | 735856007 | Injury of Achilles tendon |
| 60782007 | Pseudogout | 699163004 | Open reduction of fracture of tibia without internal fixation |
| 62972009 | Extraction | 240004005 | Semimembranosus bursitis |
| 85224001 | Pilonidal cyst with abscess | 1771000119109 | Arthritis of wrist |
| 372938004 | Acute suppurative arthritis due to bacteria | 54329005 | Acute myocardial infarction of anterior wall |
| 446403000 | Incision and drainage of abscess of skin | 76934002 | Repair of knee collateral ligaments |
| 403190006 | First degree burn | 74580009 | Cauterisation of lesion of cervix |
| 387802007 | Thoracic spondylosis | 442085002 | Greenstick fracture |
| 61486003 | Sacral back pain | 239733006 | Anterior knee pain |
| 301717006 | Right upper quadrant pain | 283165009 | Decompression laminectomy |
| 194734000 | Mitral stenosis and aortic insufficiency | 3421000 | Open fracture of orbital floor (blow-out) |
| 274663001 | Acute pain | 121021000119105 | New daily persistent headache |
| 86406008 | HIV infection | 72011007 | Rheumatic aortic stenosis |
| 384641003 | Repair of mitral valve | 262761008 | Radiation middle ear effusion |
| 87513003 | Herpes zoster ophthalmicus | 283380005 | laceration of perineum |
| 274667000 | Jaw pain | 200635001 | Finger web space infection |
| 53406005 | Juvenile osteochondrosis of spine | 235461007 | Drainage of liver abscess |
| 282752000 | Injury of eye region | 202329003 | Detachment of the glenoid labrum and/or capsule of the shoulder joint |
| 7180009 | Meningitis | 84116009 | Compression injury of nerve |
| 88998003 | Osteophyte | 200727009 | Scalp infection |
| 78516000 | Fracture of sternum | 120001005 | Testis excision |
| 300955002 | Pain in thumb | 249297006 | Spasm of bladder |
| 95668009 | Pain in face | 202757008 | Cervical disc disorder with radiculopathy |
| 63513005 | Arthrodesis of ankle | 28626004 | Vesicocolic fistula |
| 194997002 | Pulmonary stenosis, non-rheumatic | 7927006 | Periorbital haematoma |
| 18876004 | Pain in finger | 15803009 | Bladder pain |
| 282766005 | Lower back injury | 238264002 | Drainage of pelvic abscess |
| 23346002 | Sunburn | 102831002 | Renal angle pain |
| 23714000 | Nasal vestibulitis | 285375003 | Pain in penis |
| 50172003 | Lumbar spinal fusion | 9404003 | Orbital swelling |
| 449815008 | Haematoma of perianal region | 236002003 | Retroperitoneal haematoma |
| 277188003 | Ulnar nerve entrapment | 16250001000004107 | Fracture of shoulder |
| 47458005 | Repair of hip | 10217006 | Third degree perineal laceration |
| 58170007 | Viral meningitis | 23606001 | Tympanosclerosis |
| 15574005 | Fracture of foot | 183668008 | Total avulsion of nail plate |
| 62402000 | Revision of total hip replacement | 71057007 | Infection due to Escherichia coli |
| 302396003 | Cryotherapy to skin lesion | 75924001 | Urinary cystotomy for excision of bladder diverticulum |
| 262525000 | Chest injury | 87971000 | Closed reduction of fracture of radius |
| 118468006 | Open reduction of fracture with fixation | 38539003 | Infection by Onchocerca volvulus |
| 390833005 | Osteoporosis due to corticosteroids | 6561007 | pain in urethra |
| 91302008 | Systemic infection | 262588000 | Deep partial thickness burn |
| 119954001 | Adenoid excision | 426566004 | Central pain syndrome |
| 67624004 | abscess of Bartholin's gland | 427296003 | Thalamic infarction |
| 162051008 | Right iliac fossa pain | 128192007 | Peripheral neuritis |
| 86480004 | Open reduction of fracture | 95646004 | Cerebellar degeneration |
| 1121000119107 | Chronic neck pain | 78918006 | Open reduction of dislocation |
| 416209007 | Synovitis | 205000002 | Stenosis of bladder neck |
| 274165007 | Laceration of skin | 52577005 | Repair of auricle of ear |
| 267981009 | Pain in thoracic spine | 9267009 | Chest pain at rest |
| 302933001 | Tear of lateral meniscus of knee | 46276006 | Prolapsed haemorrhoids |
| 239868001 | Osteoarthritis of finger joint | 64662007 | Pulmonary infarction |
| 391897002 | Aspiration curettage of uterus for termination of pregnancy | 262524001 | Injury of lumbar spine |
| 239541009 | Excision of cervical intervertebral disc | 402664001 | Localised cutaneous vasculitis |
| 125596004 | Injury of elbow | 197928006 | Chronic urinary tract infection |
| 36939002 | Open reduction of fracture of femur | 81629009 | Dislocation of jaw |
| 162052001 | Left iliac fossa pain | 287019009 | Synovitis/tenosynovitis - wrist |
| 55162003 | Tooth extraction | 11296007 | Stenosis of trachea |
| 15033003 | Peritonsillar abscess | 85922006 | Fracture of hamate bone of wrist |
| 28432003 | Abscess of breast | 80247002 | Full thickness burn |
| 28944009 | Cytomegalovirus infection | 279073002 | Sore nostril |
| 274068006 | Excision of lipoma | 413102000 | Infarction of basal ganglia |
| 239732001 | Disorder of patellofemoral joint | 281814008 | Arthroscopic lateral patellar release |
| 371081002 | Arthritis of knee | 401226007 | Vertebroplasty |
| 429459001 | Arthritis of acromioclavicular joint | 88470007 | Open reduction of closed shoulder dislocation |
| 129127001 | Infection of ear | 282764008 | Injury of coccyx |
| 209557005 | Neck sprain | 406602003 | Infection due to Staphylococcus aureus |
| 31996006 | Vasculitis | 202794004 | Lumbago with sciatica |
| 309464009 | Elbow fracture | 69430001 | Abscess of vulva |
| 125615005 | Dislocation of shoulder region | 27480002 | Excision of bunion |
| 275520000 | Claudication | 95412009 | Pigmented villonodular synovitis |
| 86217007 | Avascular necrosis | 309465005 | Varicella-zoster virus infection |
| 302233006 | Renal artery stenosis | 18842008 | Corticobasal degeneration |
| 414564002 | Kyphosis deformity of spine | 285388000 | Right sided abdominal pain |
| 263247007 | Fracture of calcaneus | 359927001 | Repair of cystocele |
| 75591007 | Fracture of fibula | 14916000 | Fracture of trapezoidal bone of wrist |
| 262966007 | Rupture of muscle | 302859004 | Osteoid osteoma |
| 116290004 | Acute abdominal pain | 41137001 | Bicipital tenosynovitis |
| 70163005 | Excision of ganglion cyst | 445734009 | Fracture of body of vertebra |
| 90460009 | Neck injury | 36186002 | Polyarthropathy |
| 71620000 | Fracture of femur | 281527002 | Fracture of radial styloid |
| 239958005 | Painful arc syndrome | 250111002 | Patellar maltracking |
| 53772007 | Vitreous detachment | 59703009 | cervical spinal fusion by anterior technique |
| 18171007 | Fracture of phalanx of finger | 361156002 | Surgical repair of prolapsed uterus |
| 178156005 | Repair of tendo achilles | 11817007 | Actinomycotic infection |
| 18347007 | Spinal stenosis of lumbar region | 15694008 | Repair of spigelian hernia |
| 414293001 | Fracture of tibia & fibula | 442538002 | Stress fracture of fibula |
| 407153006 | Motor vehicle injury | 203337003 | Paget's disease-carpal bone |
| 31928004 | Abscess of skin &/OR subcutaneous tissue | 45177002 | Swelling of structure of eye |
| 127279002 | Injury of lower limb | 58075000 | Contusion of toe |
| 373945007 | Pericardial effusion | 47227006 | Excision of submandibular gland |
| 63491006 | Intermittent claudication | 116142003 | Radical hysterectomy |
| 281245003 | Musculoskeletal chest pain | 3.01E+11 | Muscle spasm of cervical muscle of neck |
| 4969004 | Sinus pain | 19921004 | Crushing injury (morphology) |
| 298672007 | Anterior decompression of shoulder joint | 450623005 | Biopsy of lesion of skin |
| 55222007 | Breast tenderness | 46694006 | Discomfort of vulva |
| 225553008 | wound dehiscence | 23382007 | Stress fracture |
| 239867006 | Osteoarthritis of wrist | 1755008 | Old myocardial infarction |
| 63643000 | Derangement of knee | 274137005 | Lumbar disc lesion |
| 430715008 | Bariatric operative procedure | 330007 | Occipital headache |
| 68653001 | Anal pain | 202928001 | Infected bunion |
| 262526004 | Wound of skin | 431709001 | Pilonidal abscess of natal cleft |
| 10679007 | Infection by Giardia lamblia | 275488008 | Sore throat - chronic |
| 428257007 | Fracture of tibial plateau | 235381007 | Repair of anal sphincter |
| 1926006 | Osteopetrosis | 263197004 | Fracture of radial neck |
| 279058003 | Neurogenic pain | 300886007 | Swelling of skin |
| 20793008 | Scapulalgia | 283543003 | Puncture wound of sole of foot |
| 70704007 | Sprain of wrist | 33931005 | injury of lip |
| 54888009 | Sprain of knee | 438505003 | Strain of trapezius muscle |
| 13695006 | Fracture of pubic rami | 297194001 | Ganglion of foot |
| 85875009 | Debridement of wound of skin | 448581008 | obstruction of ventricular outflow tract |
| 75564005 | Osteitis of pelvic region | 285348005 | Strain of abdominal muscle |
| 63398001 | Bacterial infection due to Pseudomonas | 125620005 | Dislocation of thumb |
| 43364001 | Abdominal discomfort | 119746007 | Lung excision |
| 403191005 | Second degree burn | 301913002 | Lesion of eyelid |
| 266096002 | Methicillin resistant Staphylococcus aureus infection | 274747003 | Localised swelling, mass and lump, neck |
| 249945007 | Monoparesis - leg | 72779005 | Anorectal fistula |
| 4308002 | Repetitive strain injury | 24437000 | Excision of palmar fascia |
| 88312006 | Amputation of leg through tibia and fibula | 267982002 | Pain in lumbar spine |
| 50642008 | Complex regional pain syndrome type I | 44241007 | Heart valve stenosis |
| 13331008 | Atrophy | 367423000 | Contusion of eye |
| 398878007 | Sprain of ligament | 134407002 | Chronic back pain |
| 76956004 | Amputation of finger, except thumb | 79267007 | Retinal migraine |
| 263126002 | Ligament injury | 58494002 | Arthrodesis of elbow |
| 50082000 | Open reduction of fracture of tibia with internal fixation | 283363006 | Laceration of lip |
| 193967004 | Swelling of eyelid | 312428002 | Corneal infection |
| 232209000 | Nasal obstruction | 422840005 | Mass lesion of brain |
| 238171002 | Repair of femoral hernia | 41000119109 | Strain of foot |
| 397112000 | Simple excision of pterygium | 314407005 | Retinal dystrophy |
| 371082009 | Arthritis of spine | 300995000 | Exercise-induced angina |
| 73583000 | Epicondylitis | 309710005 | Lumbosacral strain |
| 287572003 | Diagnostic aspiration of breast cyst | 399963005 | Abrasion |
| 9991008 | Abdominal colic | 289936007 | Shave excision |
| 281758003 | Repair of tendon | 432615008 | Chronic pain in face |
| 443395009 | Compression fracture | 95457000 | Brain stem infarction |
| 9682006 | Fracture of scapula | 314487008 | Anterior mesh vaginal repair |
| 127278005 | Injury of upper limb | 274668005 | Non-cardiac chest pain |
| 405773007 | Kyphoscoliosis deformity of spine | 773295006 | Ingrown toenail of right foot with infection |
| 75857000 | Fracture of radius & ulna | 444448004 | Injury of medial collateral ligament of knee |
| 125608002 | Fracture of lumbar spine | 410713007 | Sore sensation quality |
| 31487001 | Disseminated idiopathic skeletal hyperostosis | 95441000 | Pulmonary artery stenosis |
| 428297005 | Excision of cyst | 27477003 | Fracture of face bones |
| 11543004 | Otosclerosis | 20944008 | Congenital postural scoliosis |
| 263226008 | Subcapital fracture of neck of femur | 307184009 | Undisplaced fracture |
| 15802004 | Dystonia | 263199001 | Fracture of distal end of radius |
| 15018006 | Laparoscopic repair of inguinal hernia | 446295000 | Excision of scar |
| 440668008 | Replacement of implantable contraceptive capsule | 75140002 | Carpopedal spasm |
| 432504007 | Cerebral infarction | 5715200 | Repair of hallux valgus |
| 95460007 | Cerebellar infarction | 83270006 | Neoplastic pleural effusion |
| 238968009 | Vulvodynia | 416920000 | Obstruction of lacrimal canaliculus |
| 23680005 | Enthesopathy | 76670001 | Duchenne muscular dystrophy |
| 410016009 | Lipodermatosclerosis | 162068007 | Abdominal distension symptom |
| 71642004 | Fracture of skull | 415748002 | Traumatic rupture of patellar tendon |
| 125621009 | Dislocation of hip | 299331007 | Knee joint - varus deformity |
| 49605003 | Ophthalmoplegic migraine | 270490007 | Acute otitis media with effusion |
| 263196008 | Fracture of radial head | 448488004 | Deformity of chest wall |
| 423849004 | Iliotibial band friction syndrome | 441546003 | Periprosthetic fracture |
| 443435007 | Total replacement of right hip joint | 11679003 | Radicular pain |
| 239419007 | Repair of meniscus | 283387008 | Laceration of lower leg |
| 54556006 | Fracture of ulna | 233843008 | Silent myocardial infarction |
| 36948007 | Fasciitis | 30760008 | Finger clubbing |
| 8420001 | Abrasion procedure | 304542004 | nonspecific abdominal pain |
| 239792003 | Seronegative rheumatoid arthritis | 271681002 | Stomach ache |
| 203095000 | Spasm of back muscles | 197919005 | stenosis of urinary meatus |
| 125665001 | Crushing injury | 51169003 | Pneumococcal meningitis |
| 95598005 | Ruptured cyst of ovary | 6188005 | Arthrotomy of knee |
| 125593007 | Injury of face | 427020007 | Cerebral vasculitis |
| 444899003 | Pain in forearm | 72910005 | Lumbar sympathectomy |
| 443681002 | Total replacement of left knee joint | 300570005 | Perianal lump |
| 269219004 | Subungual haematoma | 127561000 | Valgus deformity |
| 95671001 | Cervical nerve root compression | 34476008 | Viral encephalitis |
| 49915006 | Tricuspid valve stenosis | 15743005 | Posttraumatic osteoporosis |
| 62647006 | Painful spasm of anus | 57152006 | Repair of hallux valgus |
| 41931001 | Abdominal distension | 247454005 | Sore on skin |
| 309087008 | Paraesthesia of foot | 299276009 | Limited systemic sclerosis |
| 281543009 | Strain of tendon of medial thigh muscle | 47212006 | Linear atrophy |
| 275496003 | Nasal infection | 402886003 | Primary herpes simplex infection of lips |
| 239838005 | Chondrocalcinosis | 55390008 | Arthrotomy of ankle |
| 430263007 | Arthroscopic shoulder decompression | 229844004 | Deformity of foot |
| 87642003 | Dislocation | 23589004 | Injury of spleen |
| 127287001 | Intertrochanteric fracture | 53332000 | spinal arthritis deformans |
| 66339000 | Arthroscopy of ankle | 134291007 | Multiple fractures |
| 12092003 | Repair of rectal prolapse | 299567001 | Deformity of toe |
| 385468004 | Cataract extraction and insertion of intraocular lens | 444849002 | Avascular necrosis of bone of hip |
| 397503006 | Salmonella enterica subspecies arizonae infection | 264558008 | Pelvic haematoma |
| 239542002 | Lumbar discectomy | 279074008 | Sore skin |
| 45170000 | Encephalitis | 282077009 | Laceration of anus |
| 162053006 | Suprapubic pain | 238426002 | Moraxella infection of skin |
| 46675001 | Osteoporotic fracture | 195647007 | Acute respiratory infections |
| 22247000 | Dehiscence of surgical wound | 202333005 | Triangular fibrocartilage tear |
| 13714004 | Arthroscopy | 274164006 | Minor head injury |
| 95415006 | Polymyalgia | 262784001 | Contusion of lung |
| 301777002 | Neck swelling | 282777004 | Injury of great toe |
| 263029007 | Dislocation of patellofemoral joint | 410379003 | Dressing change/wound care surveillance |
| 274038002 | Repair of hydrocele | 250072004 | Protective muscle spasm |
| 125600009 | Injury of hip region | 10461000 | Nonvenomous insect bite with infection |
| 360450007 | Strain of neck muscle | 201952005 | Traumatic arthropathy-knee |
| 302258001 | Back problem | 112633009 | Surgical wound |
| 262522002 | Injury of cervical spine | 425265006 | Knife wound |
| 252432008 | radionuclide myocardial perfusion study | 269406001 | Post-traumatic wound infection |
| 81996005 | Torsion of testis | 83070001 | Friction blister with infection |
| 415749005 | Rupture of tendon | 301034006 | Fracture of greater trochanter |
| 208921000 | Acute meniscal tear, lateral | 75582008 | Open reduction of dislocation of hip |
| 19578002 | Arthrodesis | 410062001 | Laceration of vagina |
| 247348008 | Tenderness | 442048005 | Tenosynovitis of wrist |
| 125872003 | Fracture of sacrum | 283386004 | Laceration of knee |
| 125610000 | Joint injury | 302129007 | Decompression of lumbar spine |
| 38716007 | Atherosclerosis | 715266002 | Infection of skin of eyelid and periocular region |
| 283396008 | Incised wound | 233970002 | Coronary artery stenosis |
| 417163006 | Traumatic &/OR non-traumatic injury | 705681006 | Burn dressing |
| 150062003 | Osteotomy | 43242008 | Drug withdrawal headache |
| 262536007 | Superficial abrasion | 77506005 | Infection by Trypanosoma cruzi |
| 432473000 | Femoral acetabular impingement | 90036004 | Vitelliform dystrophy |
| 11218009 | Infection due to Pseudomonas aeruginosa | 192781003 | Leucodystrophy |
| 41888000 | Temporomandibular joint disorder | 250087009 | Joint deformity |
| 35926005 | Repair of knee cruciate ligaments | 65935005 | Repair of stress incontinence by suprapubic sling |
| 81723002 | Amputation | 77956009 | Steinert myotonic dystrophy syndrome |
| 23513009 | Herpesvirus infection | 275321000 | Vulva sore |
| 399269003 | Arthropathy | 264082005 | Lumbar |
| 424128006 | Decompression | 265711005 | Operation on lumbar spine |
| 396332003 | Rheumatism | 298349001 | Soft tissue swelling |
| 66540002 | Strain of thoracic region | 443524000 | Secondary osteoarthritis |
| 9877001 | Repair of paraumbilical hernia | 449338000 | replacement of aortic root using pulmonary valve autograft and replacement of pulmonary valve |
| 1781000119107 | Sprain of ankle grade II | 14893008 | Burn of hand |
| 30556007 | Recurrent dislocation of shoulder region | 202888004 | Anterior shin splints |
| 122459003 | Dissection procedure | 31191000 | Excision of septum of uterus |
| 1791000119105 | Sprain of ankle grade I | 44275008 | Manipulation of spine |
| 26396009 | Subluxation | 301716002 | Left lower quadrant pain |
| 450554001 | Sutured laceration | 240219003 | Spinal claudication |
| 202708005 | Prolapsed lumbar intervertebral disc | 225562005 | Pressure sore on sacrum |
| 299037003 | Swelling of hand | 429586004 | Chlamydophila psittaci infection |
| 413181005 | Laparoscopic repair of hiatus hernia | 41413006 | Temporal headache |
| 240037007 | Tendon injury | 283497000 | Puncture wound of skin |
| 37785001 | Patellar tendonitis | 312901001 | Vitreomacular traction syndrome |
| 450521003 | Patellofemoral osteoarthritis | 22668006 | Subglottic stenosis |
| 21954000 | Herpes zoster auricularis | 367475009 | Lesion of ulnar nerve |
| 56557000 | Fibrositis | 88655004 | Repair of varicocele |
| 443682009 | Total replacement of right knee joint | 359532006 | Rotator cuff impingement syndrome |
| 278849000 | Cerebral atrophy | 431947002 | Injury of sacrum due to trauma |
| 19491003 | Injury of nose | 279028009 | Ovarian pain |
| 240589008 | Chlamydia trachomatis infection | 301775005 | Infection of uterus |
| 82991003 | Generalised aches and pains | 302964004 | Dislocation of temporomandibular joint |
| 125606003 | Fracture of cervical spine | 432249006 | Infarction of spinal cord |
| 240367005 | Dientamoeba fragilis infection | 231045009 | Decompression of spinal nerve root |
| 12584003 | Bone pain | 240021001 | Impingement syndrome of ankle |
| 79733001 | Amputation above-knee | 371568002 | Anal infection |
| 35899005 | Pott's fracture | 445247000 | Inflammation of bursa of patella |
| 77880009 | Rectal pain | 246756004 | Torsion deviation of eye |
| 122462000 | Drainage procedure | 230507009 | Retrobulbar neuritis |
| 386556002 | Repair of middle ear | 370247008 | Facial laceration |
| 274160002 | Fracture of phalanx of thumb | 263245004 | Fracture of tarsal bone |
| 107938000 | Small intestine excision | 209508007 | Strain of patellar tendon |
| 197079003 | Subacute intestinal obstruction | 44278005 | Puncture aspiration of cyst of skin |
| 20502007 | Pain in scrotum | 301012009 | Proteus urinary tract infection |
| 240871004 | Capillaria aerophila chest infection | 36899001 | Loop electrosurgical excision procedure |
| 312609001 | Puncture wound - injury | 178100006 | Aspiration of ganglion |
| 71286001 | Spinal cord compression | 283359004 | Laceration of forehead |
| 275952008 | O/E wound healing delayed | 399091004 | Facioscapulohumeral muscular dystrophy |
| 240003004 | Suprapatellar bursitis | 58188004 | Traumatic arthropathy |
| 108034003 | Bladder excision | 82117004 | Abrasion and/or friction burn with infection |
| 10836008 | Decompression of spinal cord | 249785006 | Tibial torsion |
| 95851007 | Fracture of orbit | 193030005 | Migraine variants |
| 41180005 | Excision of cyst of breast | 300569009 | Perineal lump |
| 10601006 | Pain in lower limb | 285387005 | Left sided abdominal pain |
| 441558003 | Stress fracture of metatarsal bone | 174836000 | Repair of defect of the atrioventricular septum |
| 17450006 | Myoclonus | 307391000 | Haematoma of thigh |
| 239866002 | Osteoarthritis of elbow | 425852005 | Fracture malunion |
| 162145001 | Vulval pain | 298731003 | Pain of sternum |
| 299966003 | Compression of lumbar nerve root | 58997001 | Chronic appendicitis |
| 4720007 | Dystrophy | 9579005 | Subtalar arthrodesis |
| 45231001 | Infrapatellar bursitis | 314630009 | Abscess of labia |
| 111255008 | Avascular necrosis of the capital femoral epiphysis | 263079005 | Fracture dislocation of wrist joint |
| 26538006 | Degeneration of lumbar intervertebral disc | 240107001 | Viral myalgia |
| 278528006 | Facial swelling | 120038005 | Cervix excision |
| 402469004 | Gouty tophus | 236645006 | Bladder outflow obstruction |
| 8920006 | Repair of retina for retinal detachment | 45685007 | Radical neck dissection |
| 36031001 | Burning feet | 300513000 | Lesion of penis |
| 23482006 | Avulsion fracture | 298397000 | Lesion of neck |
| 54586004 | Lower abdominal pain | 421204004 | Blastocystis hominis infection |
| 423451008 | Infection due to anaerobic bacteria | 283926005 | Rupture of urethra |
| 85189001 | Acute appendicitis | 230471006 | Chronic tension-type headache |
| 66944004 | Autoimmune thyroiditis | 248521008 | Lump on finger |
| 36778005 | Fracture of distal phalanx of finger | 178095009 | Excision of ganglion of wrist |
| 31757006 | Osteotomy of tibia | 47378001 | Viral thyroiditis |
| 263191003 | Fracture of neck of humerus | 13810000 | Congenital dislocation |
| 423466006 | Arthroscopic procedure | 35363006 | Infantile colic |
| 71408000 | Repair of ectropion | 335002 | Pylorospasm |
| 30473006 | Pelvic pain | 262541004 | Superficial laceration |
| 445429009 | Cervical laminectomy | 102482005 | Growing pains |
| 59292006 | Hemiplegic migraine | 40658002 | Amputation of forearm through radius & ulna |
| 297142003 | Foot swelling | 370612006 | Excision of neoplasm |
| 298012000 | Wound pain | 48333001 | Burn injury |
| 30753002 | Normal pressure hydrocephalus | 235055003 | Oral herpes simplex infection |
| 281449006 | Loosening of unicondylar knee replacement | 247352008 | Subcostal pain |
| 60937000 | Degeneration of lumbosacral intervertebral disc | 360437006 | Strain of tendon of neck |
| 301890004 | Excision of skin cyst | 105618004 | Contusion with intact skin |
| 72704001 | Fracture | 402938009 | Staphylococcal infection of skin |
| 36202009 | Fracture of tooth | 36163009 | Night pain |
| 86255002 | Arthroscopy of knee with medial meniscectomy | 125592002 | Injury of integument |
| 76634004 | Repair of anal fistula | 75119003 | Amoebic liver abscess |
| 363563002 | Entrapment | 285395009 | Strain of calf muscle |
| 79012001 | Vaginospasm | 22817005 | Strain of Achilles tendon |
| 88584000 | Evacuation of subungual haematoma | 416956002 | Undifferentiated inflammatory polyarthritis |
| 301350008 | Lesion of lip | 125616006 | Traumatic dislocation of clavicle |
| 440714005 | Genital Herpes simplex type 1 infection | 186431008 | Clostridium difficile infection |
| 47123000 | Rupture of cruciate ligaments | 425359009 | Blunt injury |
| 239729004 | Rupture of medial collateral ligament of knee | 263031003 | Subluxation of joint |
| 95690009 | Retinal tear | 265859004 | Repair of patent ductus arteriosus |
| 81953000 | Chest pain on exertion | 60897004 | Contusion of nose |
| 174328000 | Excision of sphincter of anus | 307176005 | Acute sciatica |
| 235774002 | Colonic diverticular abscess | 425802001 | Bilateral pleural effusion |
| 42262007 | Total shoulder replacement | 288012002 | TUR - bladder ulcer excision |
| 359554008 | Charcot's arthropathy | 202383000 | Ankle joint effusion |
| 263019000 | Dislocation of acromioclavicular joint | 230481005 | Idiopathic stabbing headache |
| 202942009 | Ganglion of wrist | 35908007 | Chronic arthritis |
| 42452002 | Thoracic radiculopathy | 35611005 | Rebound tenderness |
| 239164002 | Wound discharge | 76886005 | Polyneuritis |
| 27601005 | Helminth infection | 301754002 | Right lower quadrant pain |
| 299060006 | Swelling of finger | 37053006 | Open reduction of fracture of humerus with internal fixation |
| 16631009 | Transverse myelopathy syndrome | 426002008 | Infected olecranon bursa |
| 27635008 | Aching pain | 134194006 | Delayed union of fracture |
| 399165002 | Burning mouth syndrome | 300457003 | Lesion of bladder |
| 42942008 | Compression fracture of vertebral column | 69163003 | Taenia saginata infection |
| 367403001 | Pyloric stenosis | 402403000 | Urticaria secondary to infection |
| 68449006 | Arthritis of hip | 24063002 | Fracture of base of skull |
| 699207005 | Arthritis of pelvis | 209301000 | Fracture dislocation/subluxation finger/thumb |
| 63198006 | Chondromalacia | 102626001 | Liver pain |
| 274056002 | Arthrodesis of knee | 5262007 | Spinal muscular atrophy |
| 302809008 | Streptococcus pyogenes infection | 50818007 | Painful ejaculation |
| 300331000 | Lesion of liver | 29838200 | Scoliosis deformity of spine |
| 20022000 | Hemiparesis | 263042007 | Subluxation of atlantoaxial joint |
| 179893007 | Arthroscopic acromioplasty | 64063003 | Radical cystectomy |
| 289474006 | Lesion of vulva | 279063004 | Lumbar facet joint pain |
| 299990002 | Infection of foot | 203438009 | Vertebral osteoporosis |
| 397823004 | Posterior repair of vagina | 209548004 | Sprain, lumbosacral ligament |
| 266249003 | Ventricular hypertrophy | 45613006 | Contusion of lower leg |
| 443543000 | Replacement of left knee joint | 135895003 | Epiphyseal fracture |
| 247355005 | Flank pain | 63186001 | Radical mastoidectomy |
| 58320001 | Dislocation of knee | 57829009 | Stenosis of vagina |
| 429377005 | Lumbar laminectomy | 202246002 | Recurrent dislocation of the patellofemoral joint |
| 267889007 | Generalised osteoarthritis of the hand | 300927001 | Episiotomy infection |
| 762305005 | Degeneration of spine | 290097008 | Lesion of nipple |
| 203082005 | Fibromyalgia | 196754001 | Gastric spasm |
| 48387007 | Incision of trachea | 297193007 | Ganglion of hand |
| 284196006 | Burn of skin | 247350000 | Sore nipple |
| 102613000 | Localised abdominal pain | 439216001 | Aspiration of cyst of thyroid |
| 287045000 | Pain in left arm | 32402008 | Facial spasm |
| 161973001 | Anterior chest wall pain | 431309003 | Acute urinary tract infection |
| 78435003 | Ganglion of joint | 449823005 | Tenosynovitis of thumb |
| 225359006 | Pressure area care | 95725002 | Corneal laceration |
| 72893007 | Brachial neuritis | 102615007 | Ulcer-type pain |
| 23687008 | Coronary artery spasm | 239160006 | Wound haematoma |
| 281528007 | Fracture of olecranon | 10082001 | Progressive rubella panencephalitis |
| 444735002 | Instability of pelvic floor | 7415003 | Abscess of ovary |
| 49309002 | Total ankle replacement | 234647001 | Repair of cleft lip |
| 2304001 | Discitis | 2415007 | Lumbosacral radiculopathy |
| 300035001 | Injection for plantar fasciitis | 95834000 | facial hemiatrophy |
| 714507001 | Infection of tooth socket | 46504007 | Arthrodesis of metacarpophalangeal joint |
| 297130008 | Fracture of proximal phalanx of finger | 250128004 | Small joint arthritis |
| 202664003 | Cervical myelopathy | 699694000 | chronic post-concussion headache |
| 54441004 | Fracture of shaft of femur | 239285007 | Primary open reduction of fracture of neck of femur and open fixation using dynamic hip screw |
| 416991005 | Bladder outlet obstruction | 59708000 | Multiple epiphyseal dysplasia |
| 84299009 | Neuritis | 160413008 | FH: Rheumatoid arthritis |
| 102591006 | Chest wall tenderness | 271576001 | Galeazzi fracture dislocation |
| 300246005 | Lesion of tongue | 37502002 | Rupture of rectum |
| 402121009 | Epstein-Barr virus infection | 239791005 | Seropositive rheumatoid arthritis |
| 298004008 | Lesion of nasal mucosa | 284551006 | Laceration of foot |
| 238184006 | Repair of epigastric hernia | 27204001 | Fracture |
| 239993003 | Ischial bursitis | 405545007 | Mesenteric artery stenosis |
| 69257001 | Open reduction of fracture of fibula | 263128001 | Sprain of ligament of elbow |
| 54885007 | Extraction of cataract | 173358003 | Excision of lesion of tongue |
| 65966004 | Fracture of forearm | 440375002 | Open reduction of fracture of rib |
| 95808008 | Infection of ear lobe | 14152002 | Intravenous infusion |
| 302231008 | Salmonella infection | 417607009 | Neuroinvasive St. Louis encephalitis virus infection |
| 239780003 | Arthritis due to viral infection | 162046002 | Central abdominal pain |
| 59603003 | Phantom limb syndrome | 32595002 | Mononeuritis |
| 46070005 | Repair of colostomy | 95854004 | Pulled elbow |
| 301715003 | Left upper quadrant pain | 248315005 | Lipoatrophy |
| 443658004 | Replacement of right knee joint | 65761003 | Inflammatory pain |
| 48334007 | Congenital dislocation of hip | 277890004 | Toe swelling |
| 263156006 | Fracture of maxilla | 8414002 | Abscess of nose |
| 17433009 | Ruptured ectopic pregnancy | 81211007 | Primary lateral sclerosis |
| 90584004 | Spinal cord injury | 197232005 | Anorectal pain |
| 430886005 | Disorder of vertebra | 429190007 | Recurrent dislocation of hip |
| 107963000 | Liver excision | 13793006 | Extracapsular extraction of lens |
| 300953009 | Pain in axilla | 125163009 | Healing fracture |
| 1126007 | Knee locking | 38850007 | Chronic arthropathy |
| 268029009 | Pathological fracture | 429722003 | Strain of flexor muscle of hip |
| 6654000 | Acquired hallux rigidus | 239563000 | Excision of vertebral body |
| 225565007 | Perineal pain | 40257000 | Contusion of shoulder region |
| 275319005 | Swollen legs | 209627008 | Complete tear, knee, medial collateral ligament |
| 192644005 | Meningococcal meningitis | 8640600 | Human immunodeficiency virus infection |
| 36046008 | Ischiorectal abscess | 720537002 | Meningitis B vaccination |
| 125607007 | Fracture of thoracic spine | 403946000 | Paget's disease of nipple |
| 13617004 | Tracheobronchitis | 233026009 | Excision of left atrial myxoma |
| 410795001 | Juvenile rheumatoid arthritis | 238175006 | Repair of supraumbilical hernia |
| 271613005 | Parotid swelling | 200956002 | Psoriatic arthritis with spine involvement |
| 384723003 | Radical mastectomy | 37205004 | Superficial wound |
| 198108005 | Breast infection | 448355005 | Greenstick fracture of distal radius |
| 312148000 | Fungal ear infection | 63000007 | Spasmodic cough |
| 238888007 | Fat necrosis | 447094002 | Alkaline chemical burn of cornea |
| 125619004 | Dislocation of finger | 300346007 | Lesion of gallbladder |
| 443093007 | Osteochondroma | 34430009 | Rupture of uterus |
| 182641004 | Injection of carpal tunnel | 420025004 | Non-accidental injury |
| 7430007 | Cryotherapy of skin lesion with liquid nitrogen | 95799000 | Scleral laceration |
| 73063007 | Colicky pain | 427653003 | Pain radiating to right leg |
| 74146001 | Repair of bladder | 303697006 | CT of lumbar region |
| 285385002 | Left sided chest pain | 309090002 | Infection of penis |
| 82562007 | Osteochondritis dissecans | 237371007 | Wide local excision of breast lesion |
| 174392009 | Drainage of perianal abscess | 274204004 | Corneal burn |
| 367004 | Clubbing | 430903001 | Avulsion of toenail |
| 125871005 | Fracture of coccyx | 428800008 | Contusion of soft tissue |
| 179407007 | Uncemented unicompartmental knee replacement | 12708000 | Excision of accessory nipple |
| 231839000 | Complete obstruction of lacrimal canaliculus | 135850005 | Stitch infection |
| 386682000 | Arthrodesis of foot | 287018001 | Synovitis/tenosynovitis - elbow |
| 262521009 | Spinal injury | 70422006 | acute subendocardial infarction |
| 85813009 | Open reduction of fracture of humerus | 284554003 | Avulsion - injury |
| 240033006 | Postcalcaneal bursitis | 387684008 | Transmetacarpal amputation |
| 236647003 | Urethral stenosis | 298253002 | Cervical facet joint pain |
| 281598004 | Sprain of spinal ligament | 34187009 | Severe myopia |
| 129139009 | Disorder of lumbar spine | 55860008 | Stenosis of stomach |
| 232407000 | Pharyngeal spasm | 11049006 | Cervical radiculitis |
| 440258006 | Excision of skin | 37660004 | Subdural abscess |
| 125597008 | Injury of forearm | 197926005 | Postoperative urinary tract infection |
| 387736007 | Fine needle aspiration of breast | 239817004 | Synovitis of knee |
| 27916005 | Abscess of liver | 43829003 | Chronic osteoarthritis |
| 238034001 | Repair of vaginal wall prolapse | 232562006 | Excision of vocal cord nodule |
| 13543005 | Pressure | 177854007 | Simple excision of inguinal hernial sac |
| 16046003 | Muscle rigidity | 450535001 | Open contusion |
| 238192002 | Repair of ventral hernia | 162211001 | Viral headache |
| 249944006 | Monoparesis - arm | 426933007 | Streptococcus agalactiae infection |
| 439470001 | Arteriovenous fistula | 283370006 | Laceration of elbow |
| 281792000 | Swollen calf | 202945006 | Ganglion of ankle |
| 128494003 | Rupture of skeletal muscle | 733261003 | Strain of finger |
| 125617002 | Dislocation of elbow | 58250006 | Scalding pain on urination |
| 239446002 | Stabilisation of patellofemoral joint | 289725009 | Hypertonic contractions |
| 34584009 | Tooth extraction, complete lower | 238605006 | Psoriatic nail dystrophy |
| 81099000 | Cervical arthrodesis | 284003005 | Bone injury |
| 75879005 | Abdominal migraine | 3345002 | Idiopathic osteoporosis |
| 36349006 | Burning pain | 68142008 | Contusion of upper limb |
| 86380000 | Acquired claw toes | 208434009 | Closed fracture thumb proximal phalanx |
| 127295002 | Traumatic brain injury | 262582004 | Burn of face |
| 274193006 | Superficial injury of finger | 44283002 | Abscess of tongue |
| 58126003 | Postoperative wound infection | 22878006 | Contusion of knee |
| 274279008 | Renal pain | 102619001 | Pain in oesophagus |
| 1033009 | Thoracic arthritis | 371807002 | Atypical angina |
| 263248002 | Fracture of navicular | 387647006 | Repair of ankle |
| 102556003 | Pain in upper limb | 262998001 | Sprain of toe joint |
| 128351009 | Eye infection | 274664007 | Chest pain on breathing |
| 86128003 | Rupture of tendon of biceps, long head | 34248003 | Rheumatic inflammation |
| 239862000 | Idiopathic osteoarthritis | 443874006 | Infection of tick bite |
| 399750009 | Excision of thymus | 421512008 | Nonspecific exanthematous viral infection |
| 240215009 | prolapsed cervical intervertebral disc | 211332006 | Abrasion, knee |
| 231841004 | Stenosis of nasolacrimal duct | 301410009 | Tenderness of right iliac fossa |
| 27182002 | Sprain of acromioclavicular ligament | 282779001 | Injury of little toe |
| 57182000 | Nerve injury | 239869009 | Osteoarthritis of distal interphalangeal joint |
| 399072004 | Bladder neck obstruction | 73120006 | Stenosis of duodenum |
| 439469002 | Recurrent abdominal pain | 80437003 | Abscess of abdominal wall |
| 7585008 | Fracture of triquetral bone of wrist | 397181002 | Open fracture |
| 83612000 | Osteoma | 301864002 | Transient synovitis of hip |
| 30833006 | vulvar vestibulitis | 239245001 | Transfer of patellar tendon |
| 242056005 | Accidental injury | 162048001 | Right subcostal pain |
| 177866001 | Bilateral inguinal hernia repair | 25427000 | Multiple fractures of both lower limbs |
| 50442003 | Palindromic rheumatism | 235795007 | Intersphincteric abscess |
| 718539004 | Injury of rotator cuff | 720363008 | Arthritis of lumbosacral spine |
| 263049003 | Subluxation of acromioclavicular joint | 387638003 | Painful swelling of joint |
| 263122000 | Injury of glenoid labrum of shoulder joint | 426055002 | Spinal arachnoiditis |
| 275334004 | Shoulder strain | 125602001 | Injury of lower leg |
| 2.96E+14 | Derangement of medial meniscus due to injury of knee | 8566005 | Urinary tract infection |
| 38727009 | Subacute thyroiditis | 230465000 | Migraine aura without headache |
| 61974008 | Epidural abscess | 24321005 | Fungal meningitis |
| 9292007 | Oesophageal hiatus hernia repair | 720745005 | Treatment for osteoporosis |
| 54635001 | Scalding injury | 24257003 | Injury of abdominal wall |
| 58992007 | Patellectomy | 385536005 | Hot water burn |
| 721730009 | Infection caused by Helicobacter pylori | 281359008 | Traumatic synovitis |
| 274000001 | Decompression of ulnar nerve | 50890004 | Fracture of shaft of humerus |
| 12731000 | Cervical sympathetic dystrophy | 417180005 | Undifferentiated inflammatory arthritis |
| 154283005 | Pulmonary tuberculosis | 212400009 | Scalp injury |
| 240221008 | Spondylolysis | 283388003 | Laceration of shin |
| 297131007 | Fracture of middle phalanx of finger | 72093006 | Poliomyelitis vaccination |
| 7163005 | Urinary tract obstruction | 262557004 | Deep wound |
| 398211002 | Excision of palmar aponeurosis for Dupuytren's contracture of hand | 38135000 | Decompression of nerve |
| 571000119103 | Daily headache | 302073009 | Tenderness of thyroid |
| 871000119100 | Lesion of scalp | 240104008 | Congenital myotonic dystrophy |
| 95575002 | Obstruction of pelviureteric junction | 279044000 | Total body pain syndrome |
| 52772002 | Postpartum thyroiditis | 428088000 | Laceration of head |
| 12247531000119106 | Bilateral lower leg pain | 202919002 | Tibialis posterior tenosynovitis |
| 230810008 | Excision of tumour of brain meninges | 283949007 | Laceration of penis |
| 73297009 | Muscular dystrophy | 90673000 | Burning sensation |
| 105606008 | Injury of musculoskeletal system | 274280006 | Ureteric pain |
| 609495006 | Sprain of ankle grade III | 238382001 | Wound abscess |
| 240019006 | Lesion of ligaments of the ankle region | 202913001 | Flexor tenosynovitis of thumb |
| 410502007 | Juvenile idiopathic arthritis | 204368006 | Subaortic stenosis |
| 403192003 | Third degree burn | 109297005 | Acute bursitis |
| 609362004 | Conjunctival abrasion | 128937004 | Viral infection of skin |
| 31097004 | Post poliomyelitis syndrome | 26312200 | Injury of glenoid labrum of shoulder joint |
| 708919000 | Robot assisted laparoscopic radical prostatectomy | 426555006 | Pain radiating to jaw |
| 262587005 | Superficial partial thickness burn | 397142002 | Repair of testis |
| 263229001 | Subtrochanteric fracture of femur | 122482001 | Infected bursa |
| 387637008 | Effusion of joint | 34359005 | juvenile osteochondrosis of tarsal navicular |
| 83351003 | Basilar migraine | 55033002 | Muscle contracture |
| 76948002 | Severe pain | 82423001 | Chronic pain |
| 59026006 | Blepharospasm | 12771005 | Partial excision of patella |
| 197659005 | Atrophy of kidney | 209507002 | Strain of quadriceps tendon |
| 52486002 | Necrotising fasciitis | 28836005 | Fracture impacted |
| 88092000 | Muscle atrophy | 85840009 | Epiphysitis |
| 372109003 | Disorder of joint of spine | 91943004 | Arthralgia of temporomandibular joint |
| 398199007 | Aseptic necrosis of bone | 300023000 | Arthrodesis of finger |
| 19354008 | Ganglion of tendon sheath | 449615005 | Swelling of lower leg |
| 271771009 | Joint swelling | 203389008 | Juvenile osteochondrosis of the secondary patellar centre |
| 23406007 | Fracture of upper limb | 447044003 | Subungual abscess |
| 105611005 | Sprain of joint | 31399009 | Abscess of scalp |
| 287046004 | Pain in right arm | 275273001 | Graft Infection |
| 79567008 | Incision of anal fistula | 44983007 | Intervertebral disc disorder with myelopathy |
| 238408000 | Infection of nail | 62695002 | Acute anteroseptal myocardial infarction |
| 192970008 | Cauda equina syndrome | 283355005 | Laceration of oral cavity |
| 112222000 | Raised intraocular pressure | 5900006 | Haemophilus influenzae meningitis |
| 281254000 | Large bowel obstruction | 417750006 | Conjunctival laceration |
| 282775007 | Calf injury | 279083003 | Discomfort in mouth |
| 209565008 | Lumbar sprain | 262972007 | Tendon strain |
| 237462004 | Nipple infection | 237439001 | Abscess of nipple |
| 301232003 | Lesion of lung | 9046000 | Neck injury |
| 13595002 | Torsion of ovary | 50982003 | Infection by Ascaris lumbricoides |
| 406575008 | infection due to vancomycin resistant enterococcus | 68859000 | Spondylosis without myelopathy |
| 444463001 | Bilateral replacement of knee joints | 286623009 | Laceration of tongue |
| 73452002 | Abscess of lung | 178158006 | Repair of patellar tendon |
| 111910009 | Amoebic infection | 34622000 | Temporomandibular subluxation |
| 21708004 | Osteosarcoma | 446896007 | Laceration of lower lip |
| 307138004 | Spondylolisthesis L5/S1 level | 9661000 | Fracture, healed |
| 57048009 | Contracture | 66189004 | Postmyocardial infarction syndrome |
| 287048003 | Pain in right leg | 263722006 | Complete rupture |
| 41659003 | Haemophilus infection | 202482009 | Wrist joint pain |
| 1.071E+12 | Oral lesion | 443137009 | infection of pierced pinna |
| 271719001 | Raised intracranial pressure | 10380004 | Crushing injury of finger |
| 13753008 | Hemifacial spasm | 84135009 | Ruptured abscess |
| 111367007 | Pelvic abscess | 279072007 | Sore lip |
| 225564006 | Pain of nose | 266257000 | Transient ischaemic attack |
| 186748004 | parvovirus infection | 53741008 | Coronary arteriosclerosis |
| 202373004 | Elbow joint effusion | 60573004 | Aortic valve stenosis |
| 237401009 | Drainage of breast abscess | 281897000 | Helicobacter eradication therapy |
| 84445001 | Joint stiffness | 60728008 | Swollen abdomen |
| 87343002 | Prinzmetal angina | 55827005 | Left ventricular hypertrophy |
| 608830002 | Discogenic pain | 67754003 | Aortic valve sclerosis |
| 263059002 | Subluxation of patellofemoral joint | 64586002 | Carotid artery stenosis |
| 423125000 | Closed fracture | 233370007 | Aortic aneurysm repair |
| 48145001 | Obstruction of Eustachian tube | 253676001 | Postductal aortic stenosis |
| 95421005 | Intercostal myalgia | 43878008 | Streptococcal sore throat |
| 30233002 | Swallowing painful | 74107003 | Acromegaly |
| 307131005 | Mallory-Weiss tear | 79619009 | Mitral valve stenosis |
| 287047008 | Pain in left leg | 408381007 | Migraine prophylaxis |
| 230297002 | Multiple system atrophy | 23685000 | Rheumatic heart disease |
| 275249009 | Correction of scoliosis | 195126007 | Atrial hypertrophy |
| 30374004 | Excision of uvula | 56786000 | Pulmonic valve stenosis |
| 398243002 | Repair of rectocele | 26212005 | Replacement of aortic valve |
| 112727005 | Revision of hip replacement | 34068001 | Heart valve replacement |
| 429696002 | Patellar instability | 300921000 | Subclavian artery stenosis |
| 237067000 | Chronic pelvic pain of female | 443502000 | Coronary atherosclerosis |
| 10087007 | Infection by Schistosoma | 175385008 | Excision of aneurysm of cerebral artery |
| 2999009 | Injury of ear | 46689006 | Hypertrophy of tonsils |
| 71393004 | Soreness | 230729006 | Carotid artery dissection |
| 219359001 | injury of unknown intent by electrocution | 46706006 | Replacement of intrauterine contraceptive device |
| 300879004 | Swelling of salivary gland | 123715003 | Ventricular septal defect repaired |
| 35933005 | Laceration | 248491001 | Swollen knee |
| 284002000 | Injury of dental structures | 233969003 | Superficial femoral artery stenosis |
| 234890003 | Repair of cleft palate | 303081002 | Neurogenic claudication |
| 84172003 | Spondylitis | 11822401000119108 | Anterior to posterior tear of superior glenoid labrum of left shoulder |
| 52582003 | Arthrodesis of wrist joint | 308546005 | Dissection of aorta |
| 128209004 | Chronic inflammatory demyelinating polyradiculoneuropathy | 194984004 | Aortic stenosis, non-rheumatic |
| 26650005 | Acute tracheitis | 90520006 | Vertebral artery stenosis |
| 444470001 | Injury of anterior cruciate ligament | 307999008 | Diathermy of skin lesion |
| 302395004 | Cauterisation of skin lesion | 188463006 | Chlamydial pelvic inflammatory disease |
| 258754001 | Arthroscopy of wrist | 305831000119104 | Derangement of meniscus due to injury of knee |
| 70736000 | Osteochondritis | 39400004 | Injury of liver |
| 14766002 | Aspiration | 233968006 | External iliac artery stenosis |
| 281800008 | Intravenous fluid replacement | 429196001 | Partial obstruction of small bowel |
| 283545005 | Gunshot wound | 190829000 | Chronic gouty nephropathy |
| 73105000 | Pes anserinus bursitis | 10854141000119104 | Slipped right upper capital femoral epiphysis |
| 43719000 | Structure of epiphysis | 54519002 | Basilar artery stenosis |
| 247354009 | Iliac fossa pain | 249921008 | Stiff back |
| 813001 | Ankle instability | 194990000 | Tricuspid incompetence non-rheumatic |
| 20904001 | Arthrodesis of shoulder |  |  |
