# Supplementary material for: Determining the Impact of Opioid Policy on Substance Use and Mental Health–Related Harms: Protocol for a Data Linkage Study
Source: JMIR Res Protoc. 2023 Oct 17;12:e51825. doi: 10.2196/51825 (PMC10618880; doi:10.2196/51825)
Supplement: Multimedia Appendix 2 [file resprot_v12i1e51825_app2.pdf]

IDEAS GRANTS 2020: APPLICATION ASSESSMENT SUMMARY

Applicant's Name: Suzanne Nielsen

Application ID: APP2002193

Administering Institution: Monash University

Summary of Individual Scores for your Application

The average score (1-7) provided by peer reviewers who assessed your application against each of the four assessment criteria and the final weighted average score are provided below.

Assessment Criteria (Average)	Scores for APP2002193
Research Quality (RQ) – 35%	5.500
Innovation and Creativity (I&C) – 25%	5.250
Significance (S) – 20%	5.750
Capability (C) – 20%	6.250
Weighted Average	**5.638**
Category	6

The **funding cut-off** for Ideas Grant applications was 5.588, within Category 6 (not including applications funded through structural priority funding).
