# Supplementary material for: Determining the Impact of Opioid Policy on Substance Use and Mental Health–Related Harms: Protocol for a Data Linkage Study
Source: JMIR Res Protoc. 2023 Oct 17;12:e51825. doi: 10.2196/51825 (PMC10618880; doi:10.2196/51825)
Supplement: Multimedia Appendix 3 [file resprot_v12i1e51825_app3.pdf]

Australian Government
National Health and Medical Research Council

NHMRC

IDEAS GRANTS 2020: APPLICATION ASSESSMENT SUMMARY

Applicant's Name: Suzanne Nielsen

Application ID: APP2002193

Administering Institution: Monash University

Summary of Individual Scores for your Application

The average score (1-7) provided by peer reviewers who assessed your application against each of the four assessment criteria and the final weighted average score are provided below.

Assessment Criteria (Average)	Scores for APP2002193
Research Quality (RQ) – 35%	5.500
Innovation and Creativity (I&C) – 25%	5.250
Significance (S) – 20%	5.750
Capability (C) – 20%	6.250
Weighted Average	**5.638**
Category	6

The **funding cut-off** for Ideas Grant applications was 5.588, within Category 6 (not including applications funded through structural priority funding).

Australian Government
National Health and Medical Research Council

Suzanne Nielsen
suzanne.nielsen@monash.edu

Dear Applicant

********* UNDER EMBARGO AND PROVIDED IN CONFIDENCE *********

This advice and document/s referred to below are provided under strict embargo and as such, on an in confidence basis. **The document/s and the information are not to be made public at this time by institutions or recipients**. NHMRC will notify your Administering Institution when your outcome is no longer under embargo.

****

Application ID: APP2002193
Type: Ideas Grants
Application Title: Enabling evidence-informed policy to address Australia's opioid crisis

I am pleased to advise that the Minister for Health has approved your application (APP2002193) for National Health and Medical Research Council (NHMRC) Ideas Grants funding commencing in 2021.

This letter provides you with important information about the offer of Funding made to Monash University for this application.

Assessment Details

Where available[1], information about the assessment of your application is provided in a separate Application Assessment Summary. This can be accessed via RGMS following the instructions for accessing feedback in the *RGMS User Guide – Awarding Grants*.

Accepting this offer

The offer of Funding for your Application is made under NHMRC's Funding Agreement (the Funding Agreement) between the Australian Government and your Administering Institution. Your Administering Institution is responsible for informing you about the requirements of the Funding Agreement (including its Schedules), the Direct Research Costs guidelines and other applicable policies[2].

Your Administering Institution has until 16/12/2020 to certify that the information required prior to payment being made (see below) has been entered into RGMS, and to advise NHMRC of its acceptance of the offer. If the offer is not accepted by this date it may lapse. If you wish to discuss this offer of Funding, or have any queries, please contact your Research Administration Officer (RAO).

[1] An Application Assessment Summary is not available for applications to grant opportunities where NHMRC does not perform the peer review.

[2] Funding Agreement, Direct Research Costs Guidelines and other policies are available on the Funding Agreement and Deeds of Agreement webpage.

Information required prior to payment being made

Where applicable, and except where otherwise indicated, NHMRC will temporarily withhold some or all of the Funding under subclause 15.2.a of the Funding Agreement with your Administering Institution until Specified Personnel with outstanding obligations from previous NHMRC grants, including submission of a Final Report, have met those obligations.

In some circumstances, CIAs may need to provide additional ethics information. This information must be entered into RGMS by the CIA and certified by the RAO. The *RGMS User Guide – Awarding Grants* provides details on how to enter and certify this data at award. Should you have any questions concerning the provision of such information, please speak to your RAO.

If you need to seek approval to defer the start date of this grant, please refer to the Grantee Variations webpage or speak with your RAO.

Funding

As set out in the Schedule to the Funding Agreement, the Ideas Grant APP2002193, has been awarded $607,538.10. Where applicable, this budget has been assessed by the peer review panel as sufficient to complete the aims and objectives of the research proposal stated in the application for funding. Any conditions relevant to receiving the Funding are set out in the Schedule to the Funding Agreement and, where applicable, the associated Funding Policy. All expenditure must be in accordance with the requirements of the Funding Agreement.

Participation in NHMRC Peer Review

NHMRC relies on the ongoing participation of the research community to ensure that every application receives expert peer review. NHMRC is grateful for this contribution which is acknowledged on its website's peer review honour roll.

To ensure that applications for future rounds are appropriately assessed, all Specified Personnel working on NHMRC Funded Research Activities are reminded that they may be requested to make themselves available to contribute to the peer review process, in accordance with clause 23.1 of the Funding Agreement.

Accordingly, we ask that you ensure your CV/Profile information is up to date in Sapphire to assist in the identification of appropriate peer reviewers.

Yours sincerely

[Authorised for electronic transmission]

Dr. Julie Glover
Executive Director
Research Foundations Branch
